# Substrate and flow characteristics associated with White Sturgeon recruitment in the Columbia River Basin

**DOI:** 10.1016/j.heliyon.2018.e00629

**Published:** 2018-05-21

**Authors:** J.R. Hatten, M.J. Parsley, G.J. Barton, T.R. Batt, R.L. Fosness

**Affiliations:** a U.S. Geological Survey, Western Fisheries Research Center, Seattle, WA, USA; b U.S. Geological Survey, Idaho Water Science Center, Boise, ID, USA

**Keywords:** Zoology, Environmental science, Mathematical biosciences, Hydrology, Ecology

## Abstract

A study was conducted to identify habitat characteristics associated with age 0+ White Sturgeon (*Acipenser transmontanus* Richardson, 1863) recruitment in three reaches of the Columbia River Basin: Skamania reach (consistent recruitment), John Day reach (intermittent/inconsistent recruitment), and Kootenai reach (no recruitment). Our modeling approach involved numerous steps. First, we collected information about substrate, embeddedness, and hydrodynamics in each reach. Second, we developed a set of spatially explicit predictor variables. Third, we built two habitat (probability) models with Skamania reach training data where White Sturgeon recruitment was consistent. Fourth, we created spawning maps of each reach by populating the habitat models with in-reach physical metrics (substrate, embeddedness, and hydrodynamics). Fifth, we examined model accuracy by overlaying spawning locations in Skamania and Kootenai reaches with habitat predictions obtained from probability models. Sixth, we simulated how predicted habitat changed in each reach after manipulating physical conditions to more closely match Skamania reach. Model verification confirmed White Sturgeon generally spawned in locations with higher model probabilities in Skamania and Kootenai reaches, indicating the utility of extrapolating the models. Model simulations revealed significant gains in White Sturgeon habitat in all reaches when spring flow increased, gravel/cobble composition increased, or embeddedness decreased. The habitat models appear well suited to assist managers when identifying reach-specific factors limiting White Sturgeon recruitment in the Columbia River Basin or throughout its range.

## 1 Introduction

White sturgeon (*Acipenser transmontanus* Richardson, 1863) is the largest freshwater fish in North America and is found in large river systems along the west coast. Reproducing populations are found in the Sacramento, San Joaquin, Columbia, and Fraser river basins (Hildebrand et al., 2016). White Sturgeon spawn in water flowing about 1 m/s or faster over gravel-to-boulder sized substrates in water depths greater than about 2–3 m (Parsley et al., 1993; Perrin et al., 2003; Schaffter, 1997). Historical overharvest and vast habitat changes caused by construction of dams and reservoirs and resultant river regulation have affected white sturgeon populations throughout their range, including the Nechako (McAdam et al., 2005), Snake (Bevelhimer, 2002), and San Joaquin (Jackson et al., 2016) rivers. Riverine habitats in the Columbia River Basin have been drastically altered by dams and their associated flow and water elevation regulation (Dauble et al., 2003; Hatten and Batt, 2010). Dams prevent virtually all upstream movement as white sturgeon seldom ascend the existing fishways (Parsley et al., 2007) resulting in considerable variability in population abundance and dynamics throughout the Columbia River Basin.

White Sturgeon reproduction and recruitment to age-0 (spawning success) is more common in lower Columbia River Basin reaches and less common to non-existent in reaches further upstream (Hildebrand et al., 2016; Miller et al., 1995). Some reaches have consistent spawning success across years, that is, some age-0 fish can be captured virtually every year in specific reaches, while other reaches have intermittent or consistently poor or negligible spawning success as shown by lack of age-0 fish during fall sampling (McDonald et al., 2010; Rybacki et al., 2017). In every area of the Columbia River with severely depleted White Sturgeon populations, annual spawning has been documented but age-0 or older wild juvenile fish are rarely collected, suggesting that there is high mortality during egg incubation, larval or early juvenile stages. The cause of this early mortality likely varies among areas and is due to a variety of physical and biological factors (Anders et al., 2002; Parsley et al., 2002), but substrate quality has been identified as a primary determinant of recruitment success (Koch et al., 2006; McAdam, 2011, 2012). In the Nechako River and upper Columbia River transboundary reach, White sturgeon recruitment failure started 8–15 years after flow regulation due to fine sediments covering spawning substrates (McAdam et al., 2005; McAdam, 2015). Embeddedness of white sturgeon spawning grounds is very important since incubation and rearing of early life stages within interstitial spaces of substrates has been shown to result in faster growth, gut development, swimming performance, and survival (Baker et al., 2014; Boucher et al., 2014).

Despite knowledge that there is wide variability in success of White Sturgeon recruitment to age 0 across spawning areas, there has been only a limited attempt to compare and contrast habitats among reaches or within a reach among years to improve understanding about why juvenile production is variable. In a multi-reach comparison, Parsley and Beckman (1994) found the spawning area downstream from Bonneville Dam (hereafter referred to as the Skamania Reach), the most riverine of four areas compared (i.e, Skamania, The Dalles, John Day, McNary), produces higher quality spawning habitat at contemporary flows than upriver backwatered reaches. Furthermore, Skamania reach is the only reach that consistently produces a discernable year class, while most upstream spawning areas (e.g., John Day, McNary) are inconsistent among years, and others, such as the Kootenai River, have experienced decades of White Sturgeon recruitment failure (Hildebrand et al., 2016; Rybacki et al., 2017). In the highly altered Kootenai River, spawning White Sturgeon now select areas with highest available water velocities (generally 0.8 m/s or greater) and water depths that occur under the current river discharge (Paragamian et al., 2009). However, this strategy has not produced enough progeny to maintain the population and Kootenai River White Sturgeon was listed as endangered in 1994 (U.S. Fish and Wildlife Service (USFWS), 1994).

Our primary goal was to understand why Skamania reach has consistent White Sturgeon recruitment while John Day reach has inconsistent recruitment and Kootenai reach none. To accomplish our goal, we developed a GIS database that characterized physical features of the three reaches. Specifically, we constructed substrate and embeddedness maps after conducting detailed field surveys in each reach, and created hydrodynamic simulations (estimated water depths, velocities, Froude number) using a two-dimensional (2D) hydrodynamic model to map hydraulic features from resulting flows. Last, we conducted spatially explicit habitat modeling to identify physical factors associated with successful White Sturgeon recruitment among the three river reaches. River flow has long been linked to recruitment success (Stevens and Miller, 1970; Kohlhorst et al., 1991; Parsley and Beckman, 1994; Fish, 2010), and stream velocity has been used in a White Sturgeon spawning habitat model (Parsley and Beckman, 1994). In this study we incorporate Froude number (Jowett, 1993), a dimensionless variable derived from hydrodynamic modeling and a surrogate indicator of stream power, to better compare habitat features at different scales and geographic regions. This work represents a step toward understanding underlying causes of the production of a successful year class of White Sturgeon in the Columbia River Basin. We expect that the information provided here will be useful to biologists, water managers, and others for use in determining appropriate actions for maintaining or restoring White Sturgeon populations in degraded areas.

## 2 Methods

### 2.1 Project area

**Fig. 1:**
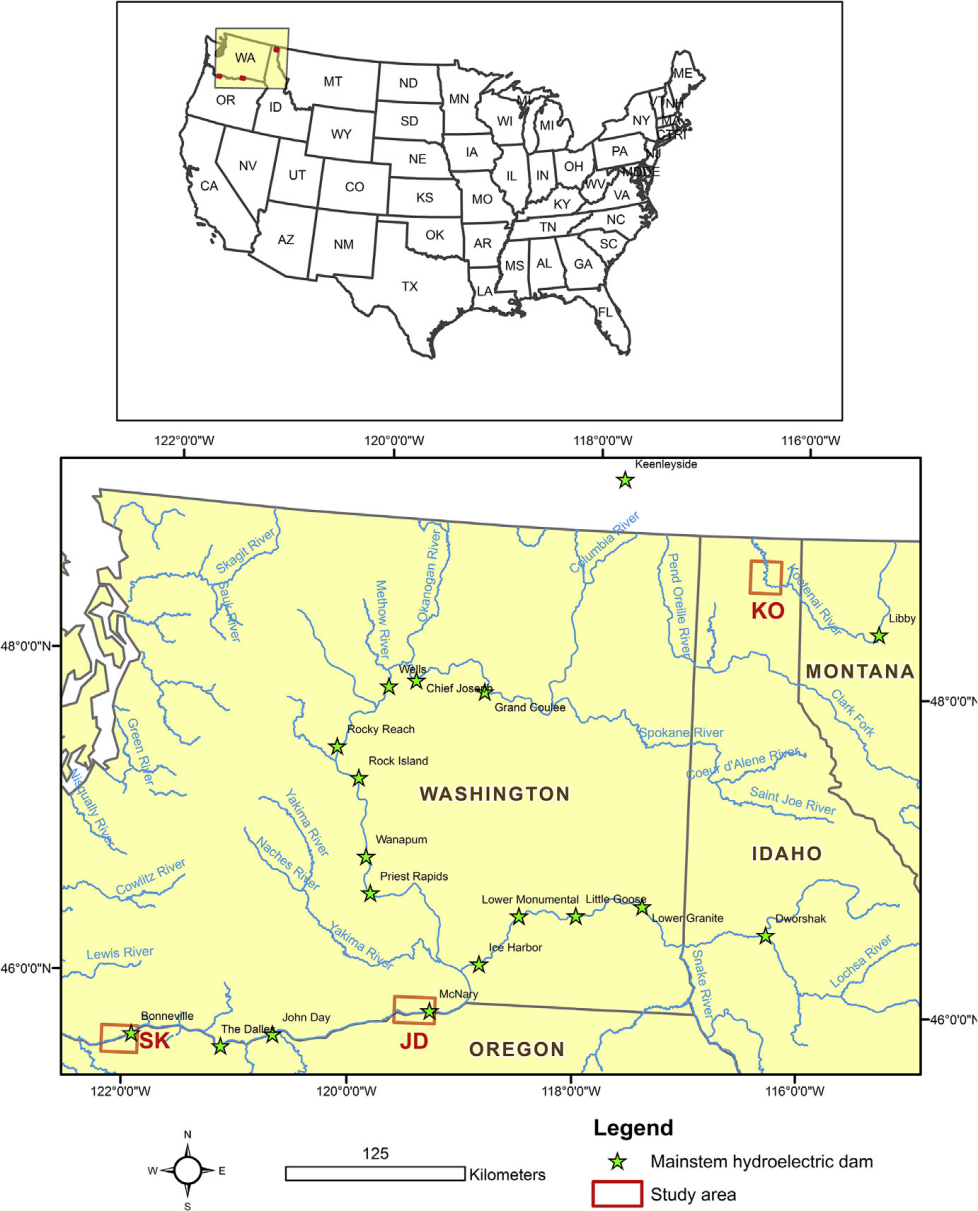
A map of the project area and the three study reaches: Skamania (SK), John Day (JD), and Kootenai (KO).

We compared physical characteristics in three reaches of the Columbia River Basin (Fig. 1). The Skamania reach extends upstream from Skamania Island (river km 217.8) to the U.S. Army Corps of Engineers boat restricted zone at Bonneville Dam (river km 232.3). The John Day reach extends from the Oregon Department of Fish and Wildlife's Irrigon Hatchery (river km 448.5) upstream to the U.S. Army Corps of Engineers boat restricted zone at McNary Dam (river km 470.0). The Kootenai (Meander) reach extends from near Ball Creek (river km 222.2) upstream to Highway 95 bridge (river km 245.9) in Bonners Ferry, ID. We framed our analyses within the last three decades because no sampling for age-0 White Sturgeon occurred prior to 1987. Although annual sampling for presence of age-0 White Sturgeon was incomplete across years in all areas, occurrence records indicated that age-0 White Sturgeon were consistently present every year in Skamania reach (Hildebrand et al., 2016), inconsistently present in John Day reach (Rybacki et al., 2017), and consistently absent in Kootenai reach (McDonald et al., 2010).

A large factor related to White Sturgeon recruitment in the Columbia River Basin is the federal hydropower system which has altered the physical conditions of each reach compared to pre-dam conditions (Parsley and Beckman, 1994; USFWS, 2008). Kootenai reach White Sturgeon appear to have been most impacted by construction of Libby Dam (1974), with a drastic reduction in the magnitude of spring flows and increased sedimentation throughout the reach (McDonald et al., 2010). White sturgeon recruitment has completely failed in Kootenai reach and most of its habitat has been designated critical habitat (USFWS, 2008). John Day reach has also been severely affected by construction of McNary (1954) and John Day (1971) dams, which decrease the magnitude of spring flows and cause significant backwatering (Parsley and Beckman, 1994). In contrast, Skamania reach has suffered the least impact from the federal hydropower system, but water storage and releases for flood control and hydropower production upstream from Bonneville Dam (1938) does reduce the magnitude of spring flows. Of the three reaches examined, only Skamania reach has no downstream impoundment and no backwatering resulting from impoundments, but it does have a small tidal influence.

### 2.2 Modeling overview

**Fig. 2:**
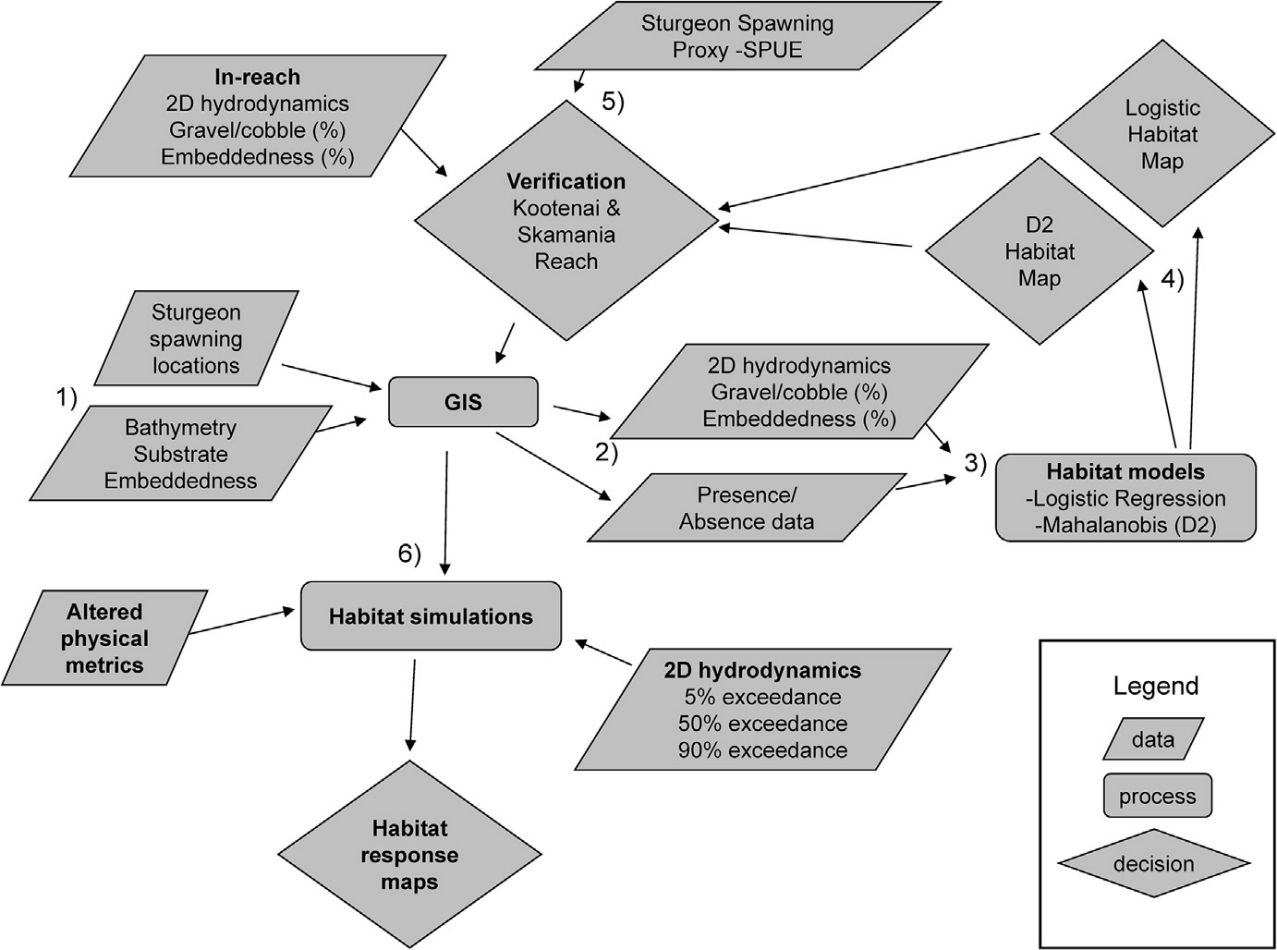
A conceptual diagram of the modeling process undertaken to characterize and map white sturgeon spawning habitat in three river reaches.

A hierarchal approach was used to achieve our goal of identifying physical factors associated with White Sturgeon recruitment (Fig. 2). First, we developed a database for habitat characterization and statistical (habitat) modeling by characterizing substrate composition, embeddedness, and hydrodynamics (depth, velocity, Froude number) of each reach in a consistent manner (Appendix 1). Second, we created a suite of predictor variables from the physical data with a geographic information system (GIS). Third, we used a dual modeling approach (Mahalanobis distance, logistic regression) to characterize and predict White Sturgeon habitat in the Skamania reach. Fourth, we created habitat maps of each reach by populating the habitat models with in-reach physical characteristics (i.e., substrate composition, hydrodynamics, embeddedness). Fifth, we assessed the accuracy of the models with spawning locations in the Skamania and Kootenai reaches. Sixth, we conducted a sensitivity analysis by simulating how predicted habitat would change if substrate and embeddedness values more closely matched the Skamania reach, populating each model with hydrodynamic data simulated at 5%, 50%, and 90% exceedance flows to determine how predicted habitat changed from high to low flows.

Variables selected for comparison within and among reaches were based on their relevance to many aspects of White Sturgeon ecology. Differences in flow volume between the Kootenai and Columbia reaches required dimensionless variables that were scale independent. We focused upon stream discharge, substrate composition, and embeddedness because they are all thought to influence spawning and recruitment success in the Columbia and Kootenai rivers (Paragamian et al., 2009; Parsley et al., 1993; Parsley and Beckman, 1994). We used Froude number in our analysis because it can identify riffle/pool habitats (Jowett, 1993) and benthic invertebrate abundance (Jowett et al., 1991), predict fish traits (Lamouroux et al., 2002), and characterize anthropogenic impacts of hydropower operations (Hatten and Batt, 2010).

**Fig. 3:**
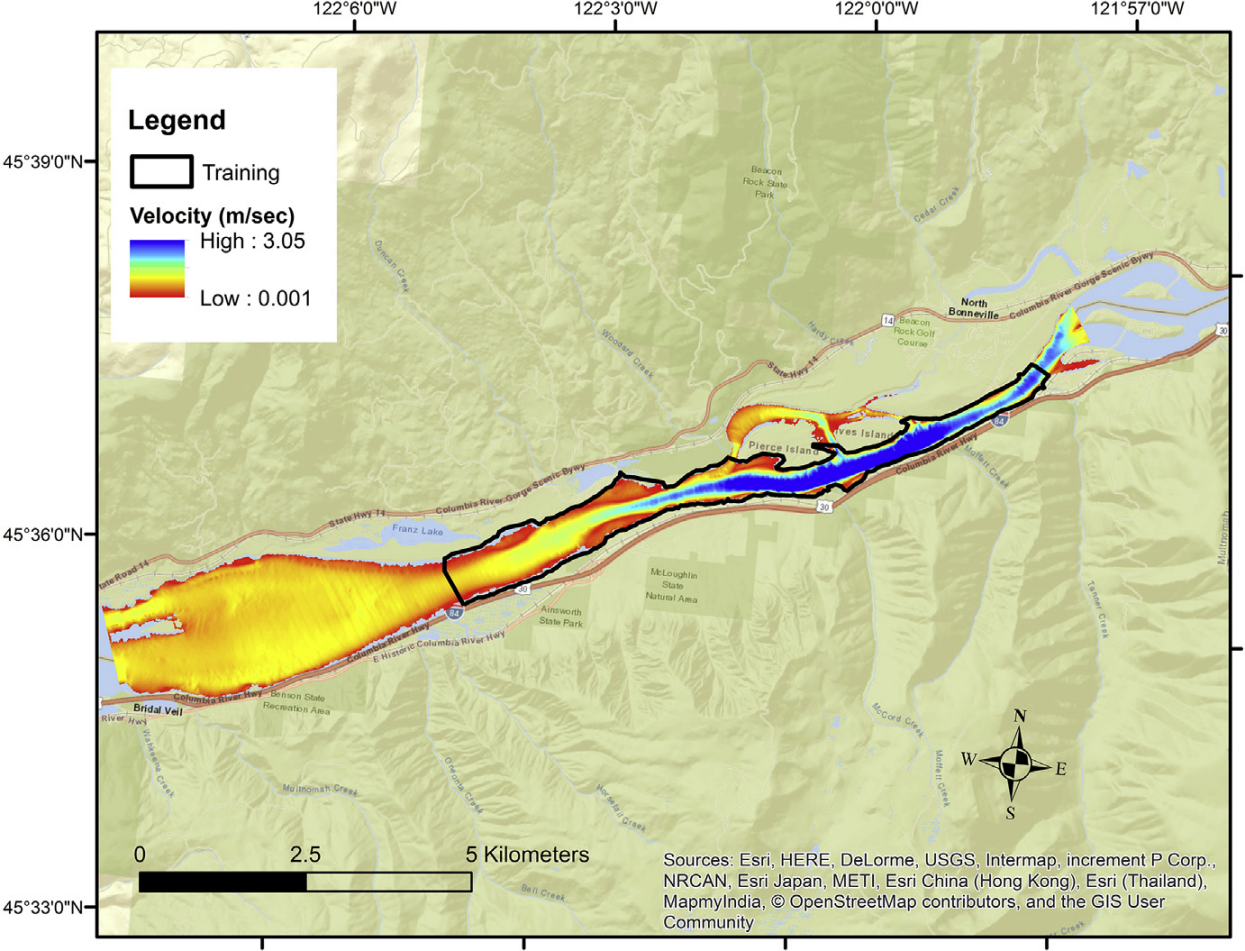
The location of the training (spawning) area (area within the dark border; RKM 223–232.3) in the Skamania reach overlaid on a velocity grid (m/sec) produced from a 2D hydrodynamic model at a 50% exceedance flow. The area outside the training area was referred to as the contrast area because it had no documented white sturgeon spawning activity.

We used a dual-modeling approach when analyzing White Sturgeon spawning habitat because it allowed us to examine our results with multiple lines of evidence (Burnham and Anderson, 2002; Johnson et al., 2017). We created a Mahalanobis distance model (Mahalanobis, 1936) since it has been used to characterize white and green sturgeon habitats in the Lower Columbia and Sacramento rivers, respectively (Hatten and Parsley, 2009; Mora et al., 2009), and logistic regression (Hosmer and Lemeshow, 2000) because of its proven flexibility to predict fish habitat at multiple spatial scales and flows in the Columbia River (Hatten et al., 2009; Tiffan et al., 2002). Mahalanobis model required presence data while the logistic model required presence/absence data. We satisfied both conditions with a spatial dataset randomly generated at locations inside and outside of a consistent White Sturgeon spawning (training) ground in Skamania reach (Fig. 3). In the following sections we provide information on (1) physical characterization of study reaches, (2) development of spatially explicit predictor variables, (3) habitat model development, (4) spatially explicit model projections, (5) model verification, and (6) sensitivity analysis.

### 2.3 Physical characterization of study reaches

Physical surveys were conducted in each reach to characterize substrate composition, embeddedness, and hydraulic conditions during spring/summer 2007 and 2008. Substrate was characterized with underwater videography throughout each reach (Appendix 1 – Mapping substrate and embeddedness). Sample locations were more dense in areas where riverbed topography varied and less dense in areas with uniform topography. Specific measurements were made of each sample to characterize substrate size classes and embeddedness levels in each location. Information was also collected related to river hydraulics using an array of echo-sounders mounted on a research vessel. Water depths, velocities, and water surface elevations were collected over a range of flows in a manner consistent with previous work conducted in Hanford (Hatten et al., 2009; Tiffan et al., 2002) and Kootenai (Barton et al., 2005; McDonald et al., 2010) reaches. Two-dimensional (2D) depth-averaged water velocities and depths were simulated for a range of flows with hydrodynamic software and calibration data obtained from the physical surveys (water surface elevations and substrate roughness coefficients).

### 2.4 Spatially explicit predictor variables

We created spatially explicit hydrodynamic, substrate, and embeddedness layers (grids) representing each reach at low (90% exceedance), moderate (50% exceedance), and high (5% exceedance) flows. Three reaches at three flows produced nine GIS layers that were rendered as grids. Spatial resolution of each layer (i.e., cell to ground distance) was 10 × 10 m (100 m^2^) for the Skamania and John Day reaches, and 5 × 5 m (25 m^2^) for the Kootenai reach. A higher spatial resolution was necessary to characterize Kootenai reach because it is an order of magnitude smaller in flow and physical dimensions (e.g., width, depth) than Skamania or John Day reaches.

We converted thematic layers (substrate and embeddedness classes) into continuous layers with a moving window since Mahalanobis model required continuous variables as input (Clark et al., 1993; Hatten and Parsley, 2009). We selected a 20-m-radius (∼0.13 ha) moving window to calculate percentage of neighborhood comprised of low to moderate embeddedness (<50% embeddedness) and percentage (area) of gravel/cobble. We selected a 20-m-radius moving window because it was small enough to characterize fine-scale riverbed details (embeddedness and substrate) while characterizing the immediate surroundings.

We produced binary grids in a three-step process. First, we used a reclassification table to recode all cells with a desired feature (i.e., gravel/cobble with low embeddedness) to a value of one, and coded the remaining cells (i.e., high embeddedness) to zero. Second, we used a moving window to count all cells within a 20-m radius that contained a target feature. Third, we divided the number of selected cells by area of the moving window (∼0.13 ha) to obtain percentage of neighborhood comprised of a target feature (e.g., 20% cobble/substrate, 15% embedded). Results of each moving-window operation were stored in grid format (cell-based) for subsequent use in Mahalanobis and logistic habitat models.

### 2.5 Development of habitat models

We developed and tested two probability models (Mahalanobis distance and logistic regression) in Skamania reach by comparing and contrasting sample locations found inside and outside of a known spawning zone (Fig. 3). Specifically, we constructed a georeferenced database for habitat modeling in several steps. We randomly selected 5,000 locations throughout Skamania reach spaced at least 20 m apart to ensure no overlap in a 20-m-radius moving window used to characterize substrate and embeddedness. Of this total, 1,881 locations occurred inside the spawning zone and 3,119 outside. We attributed sample locations with their respective zone, Froude value, gravel/cobble composition, and embeddedness values. We then randomly selected ∼80% of sample locations for model development and set the remainder (367 presences/611 absences) aside for internal (Skamania reach) model verification. We referred to sample locations inside the spawning zone as presence locations; those that occurred outside the spawning zone (hereafter “contrast zone”) were considered absence locations. We considered absence locations to be pseudo-absences since they occurred in a zone that had little spawning activity (van der Leeuw et al., 2006) but individual locations were not sampled. Our dual modeling approach (Mahalanobis and logistic) enabled us to determine the statistical contribution of pseudo absences since Mahalanobis model does not use absence locations for training while binary logistic regression does. We compared classification accuracies with internal (Skamania reach) and external (Kootenai reach) verification data. The contrast zone had islands and side channels, making it a more heterogeneous riverine environment than the training zone, which was located entirely in the swift-flowing main channel.

We used Mahalanobis distance (*D*^2^) to create a spatially explicit predictive model of White Sturgeon spawning and early life stage (recruitment) habitat because it avoids many pitfalls of statistical models and requires only presence data (Clark et al., 1993). Mahalanobis distance (*D*^2^) measures similarity obtained from the standardized squared distance between a set of sample variates and a reference condition based on the mean of variates associated with animal observations. We referred to occupied habitat as *H*, an *n* × *p* matrix of *p* variables measured at *n* locations where a sturgeon was present. We calculated a Mahalanobis distance (*D*^2^) from sample sites with the following equation (Mahalanobis, 1936):

(1) $$D^{2}\left(\text{y}\right)={\left(\text{y}-\mu \right)}^{\prime }\Sigma^{\mathrm{-1}}\left(\text{y}-\mu \right)$$

where *D*^*2*^(у) is a squared scalar distance, standardized in the Σ metric; μ is the *p* × 1 vector of mean variable values based on *H*; and y is the *p* × 1 vector of measurements at any location (it does not have to come from *H*). Thus, y – μ is a vector of deviations of a location's condition from a vector of mean habitat condition associated with White Sturgeon; Σ is the *p* × *p* variance-covariance matrix based on H. The software and code we used to implement Mahalanobis partitioning was provided by R Stats package (R Core Team, 2017).

To provide contrast with the Mahalanobis model, we developed a binary logistic regression model of White Sturgeon spawning habitat with the same predictor variables and presence locations as Mahalanobis model and a set of pseudo-absence locations (Hosmer and Lemeshow, 2000). Binary logistic regression has strong diagnostic capabilities that make it ideal for presence/absence data. We used ArcGIS (Redlands, CA) to calculate and map probability of White Sturgeon spawning habitat with the following equation:

(2) $$P=e^{g\left(x\right)}/1+e^{g\left(x\right)}$$

where: *g*(*x*) is the linear combination of parameter estimates obtained from the logistic regression. In equation (2), relative suitability of a location is linked (indexed) to the probability of spawning activity (Hatten et al., 2009). We evaluated the significance of the associations between spawning activity and substrate class, embeddedness, and Froude number with backward stepping and changes in the model's log-likelihood.

### 2.6 Spatially explicit modeling

We created a set of spatially explicit maps (grids) of potential White Sturgeon spawning habitat with Mahalanobis distance (D^2^) and logistic regression within each reach at three exceedance flows (5%, 50%, 90%) based upon underlying physical features and equations (1) or (2). We simulated effects of flow magnitude on predicted White Sturgeon habitat by populating Mahalanobis and logistic models with Froude number, embeddedness, and substrate composition, resulting in nine habitat maps per model (3 reaches X 3 flows). Only Froude number changed in these simulations since embeddedness and substrate were constants in the study area and in equations (1) or (2). The cell resolution of Skamania and John Day reaches was 10 × 10m cell-to-ground resolution, and 5 × 5m cell-to-ground resolution in Kootenai reach. The waterline obtained from the 2D hydrodynamic model at a given flow was used as the boundary of each habitat map.

### 2.7 Model verification

Model verification was a two-step process. First, we conducted verification of the Mahalanobis and logistic models in Skamania reach where they were developed with the 20% set-aside (*n* = 969), quantifying model performance across a range of binary thresholds with area under the ROC curve (AUC; Egan, 1975; Robin et al., 2011). We compared and contrasted the physical conditions and model predictions found inside the spawning area and the surrounding contrast area with box-and-whisker plots (Tukey, 1977) and a two-sample non-parametric Wilcoxon rank sum test (hereafter “Wilcoxon test”) between training (*n* = 1,596) and contrast (*n* = 2,382) locations. We then verified our models externally by projecting them to the Kootenai reach, generating binary habitat suitability maps with in-reach physical characteristics, and overlaying White Sturgeon spawning locations (McDonald et al., 2010). The Kootenai verification dataset also contained the number of White Sturgeon spawning events per unit effort (SPUE) collected between 1994 and 2002, allowing us to generate AUC at multiple SPUE thresholds.

### 2.8 Sensitivity analysis

We tested sensitivity of each habitat model by globally changing values of each predictor variable (i.e., Froude, embeddedness, gravel/cobble composition) in each reach and flow, recomputing model probabilities on a cell-by-cell basis (1,386,772 cells), and presenting results as a series of side-by-side boxplots. The focus of the sensitivity analysis was Kootenai reach since it contained the most imperiled White Sturgeon stock, but we included all reaches for comparative purposes. Specifically, we conducted three habitat simulations and compared results to baseline (contemporary) conditions in each reach at three flows. In the first simulation we globally set the gravel/cobble composition at 10% (i.e., every cell in reach was changed) and set embeddedness values at 50%. In the second simulation we globally set gravel/cobble composition at 50% and embeddedness to 10%. In the third simulation we globally set gravel/cobble composition at 90% and embeddedness to 5%. We included for reference a horizontal abline across the nine boxplots (3 flows × 3 reaches) that displayed the median model probability inside Skamania reach's training zone at a 5% exceedance flow (considered optimal), allowing one to compare model probabilities by reach, flow, and simulation. A positive result was achieved when a simulation shifted model probabilities upwards while a negative result shifted model probabilities downwards.

## 3 Results

### 3.1 Physical comparisons

**Fig. 4:**
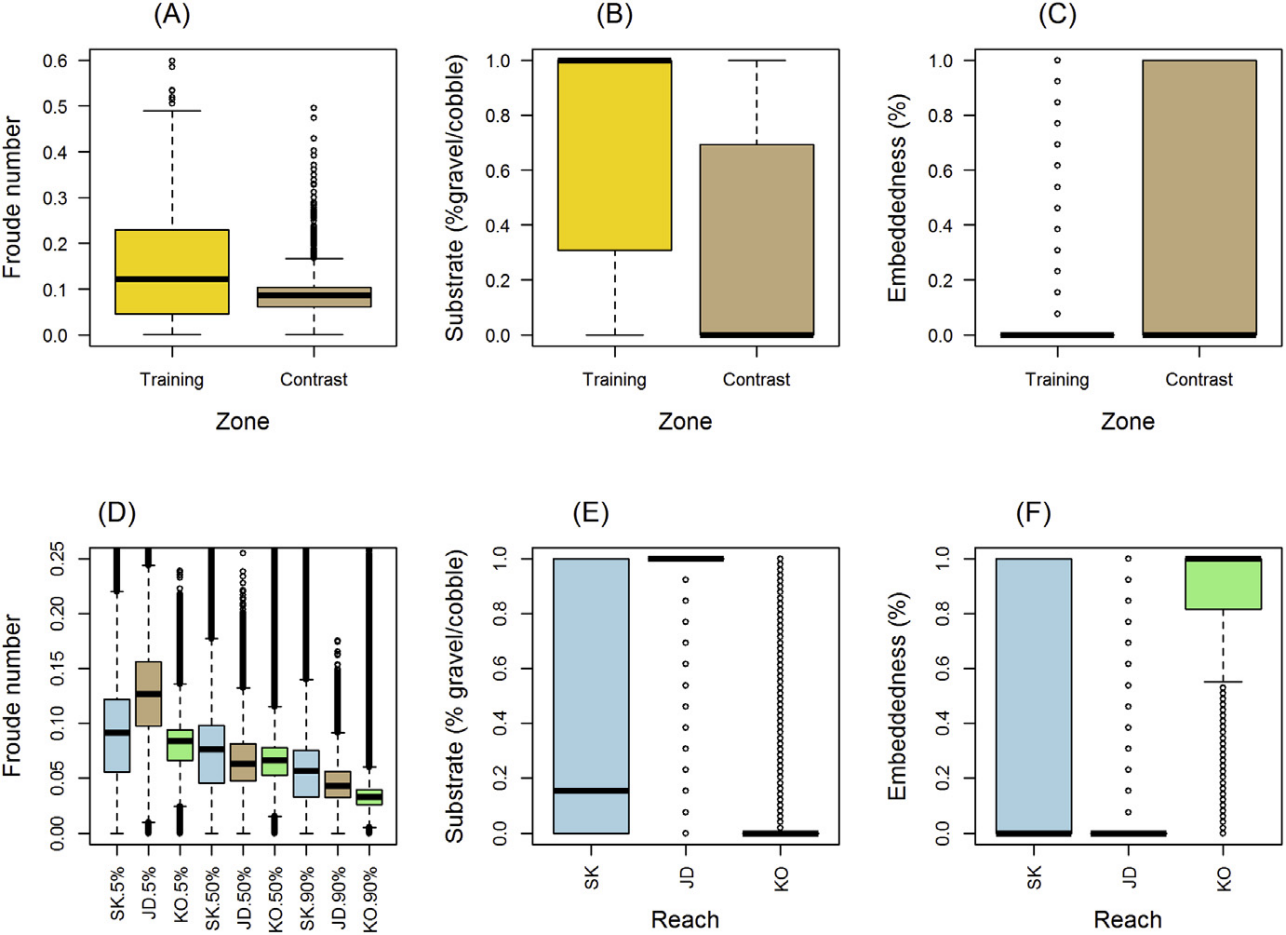
Boxplots compare Froude number (A), substrate (percent gravel/cobble) composition (B), and percent embeddedness (C) between training and contrast zones in Skamania Reach, and comparison of Froude numbers among three study reaches at three flows (D), substrate composition (E), and embeddedness (F): Skamania (SK), John Day (JD), and Kootenai (KO). Boxplots display the 25th and 75th percentiles (bottom and top of box), medians (interior horizontal line), 5th and 95th percentiles (bottom and top lines outside box), and outliers (dots).

A Wilcoxon test found differences (*P* < 0.01) in median Froude values (Fig. 4A), substrate composition (Fig. 4B), and embeddedness (Fig. 4C) between training and contrast zones for both models. Specifically, the training zone had relatively less pool habitat, lower embeddedness, and more gravel/cobble than the contrast zone, reflected by a mean Froude value of 0.146 compared with 0.091, respectively. Within the training zone, 69.1% of sample locations were pool (Froude <0.18), 29.7% glide (Froude >0.18 and <0.41), and 1.2% riffle (Froude ≥0.41), compared with 94.6% pool, 4.5% glide, and 0.8% riffle within the contrast zone. Within a 20-m radius of training locations, mean gravel/cobble composition was 70.24% compared with 27.68% in contrast locations, while mean embeddedness in the training zone was 17.24% compared with 43.31% in contrast locations. Detailed maps of each reach's physical conditions are presented in Appendix 2 (Substrate, Froude, and embeddedness maps).

Differences among the three study reaches (Wilcoxon test, *P* < 0.01) were observed between median Froude value (Fig. 4D), gravel/cobble composition (Fig. 4E), and embeddedness levels (Fig. 4F). Skamania reach's median Froude values were greater than Kootenai's, but smaller than John Day's at a 5% exceedance flow, while Skamania's Froude values were larger than John Day's or Kootenai's at 50% or 90% exceedance flows. The spread in Froude values (Fig. 4D) was largest in Skamania reach and smallest in Kootenai reach, indicating hydraulic variability was greatest in Skamania reach and smallest in Kootenai reach. Differences were observed in substrate composition and embeddedness among reaches, with Skamania reach having the largest range in conditions followed by the Kootenai reach and then the John Day reach. John Day's substrate was comprised primarily of gravel/cobble with little embeddedness, Kootenai reach contained very little gravel/cobble and was extremely embedded, while Skamania reach contained the greatest range in substrate composition and embeddedness values.

### 3.2 Habitat models

The Mahalanobis model's mean vectors and covariance matrix as obtained from training data (*n* = 1,504) and equation (1) are:

(3) $$\text{Vector}\;\text{of}\;\text{Mean}\;\text{Values}=\left(\begin{array}{l} \text{Froude}=\text{0}\text{.}146\\ \text{Substrate}=\text{0}\text{.}702\\ \text{Embeddedness}=0.172 \end{array}\right)$$

$$\text{Covariance}\;\text{Matrix}=\left(\begin{array}{ccc} \text{0}\text{.}013 & \text{0}\text{.}012 & -\text{0}\text{.}019\\ \text{0}\text{.}012 & \text{0}\text{.}175 & -\text{0}\text{.}102\\ -\text{0}\text{.}019 & -\text{0}\text{.}102 & \text{0}\text{.}129 \end{array}\right)$$

The logistic model's logit as obtained from training data (*n* = 3,978) and equation (2) is:

(4) $$\text{Phab}=-2.019+6.393\left(\text{Froude}\right)+1.808\left(\text{gravel/cobble}\;\text{\%}\right)+1.556\left(\text{embeddedness}\right)-1.526\left({\text{embeddedness}}^{2}\right),$$

where Phab = predicted White Sturgeon spawning habitat (see Table 1 for parameter estimates).

**Table 1:** Logistic regression parameters, coefficients (B), standard errors (S.E.), Wald statistic, degrees of freedom, parameter significance, odds ratio (Exp(B)), and 95% confidence intervals for odds ratio.

Parameters	B	S.E.	Wald	df	Sig.	Exp(B)	95.0% C.I. for Exp(B)	
							Lower	Upper
Froude	6.393	0.529	146.107	1	0.000	597.740	211.985	1685.464
Embeddedness	1.556	0.701	4.924	1	0.026	4.740	1.199	18.734
Substrate	1.808	0.092	382.516	1	0.000	6.101	5.090	7.313
Embeddedness_2	−1.526	0.704	4.702	1	0.030	0.217	0.055	0.864
Constant	−2.019	0.102	393.707	1	0.000	0.133		

**Fig. 5:**
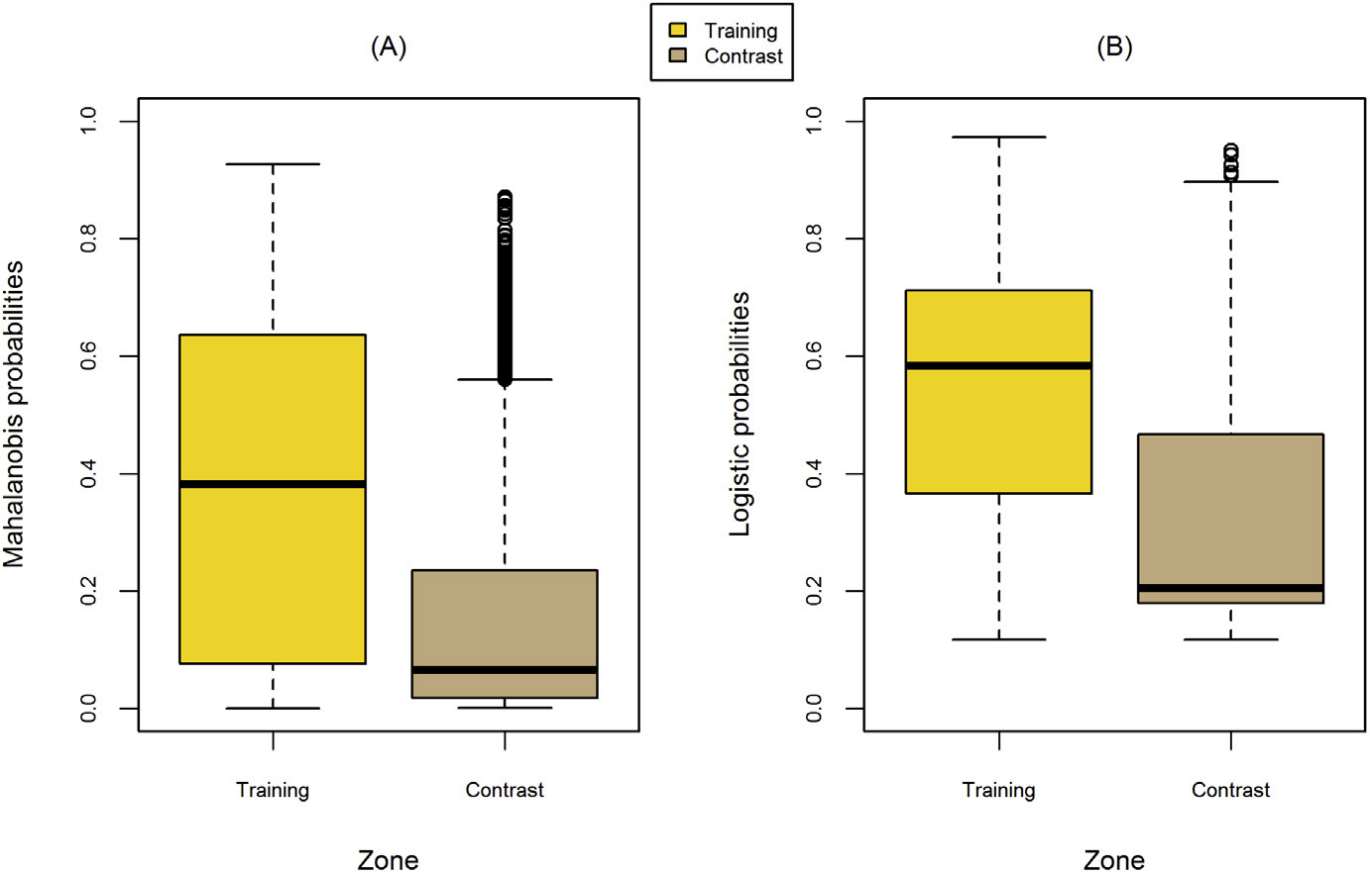
Boxplots portray the range of model probabilities in Skamania reach at a high (5% exceedance) flow obtained by Mahalanobis distance (A) and logistic regression (B) models inside the training and contrast zones (see Fig. 3). Boxplots display the 25th and 75th percentiles (bottom and top of box), medians (interior horizontal line), 5th and 95th percentiles (bottom and top lines outside box), and outliers (dots).

Boxplots revealed large differences in model probabilities between training and contrast zones, and between models. The Mahalanobis model (Fig. 5A) had a larger spread in model probabilities inside the training area than the logistic model (Fig. 5B), but a smaller range in model probabilities inside the contrast area, indicating different model sensitivities. A Wilcoxon test found all median model probabilities were different (*P* < 0.01) between zones and models. Substrate composition was the most significant predictor in the logistic model, followed by Froude number and embeddedness, respectively. A discriminant analysis of Mahalanobis' predictor variables (not shown) found the same order of significance for predictor variables as the logistic model obtained through backward stepping.

### 3.3 Spatially explicit habitat modeling

**Fig. 6:**
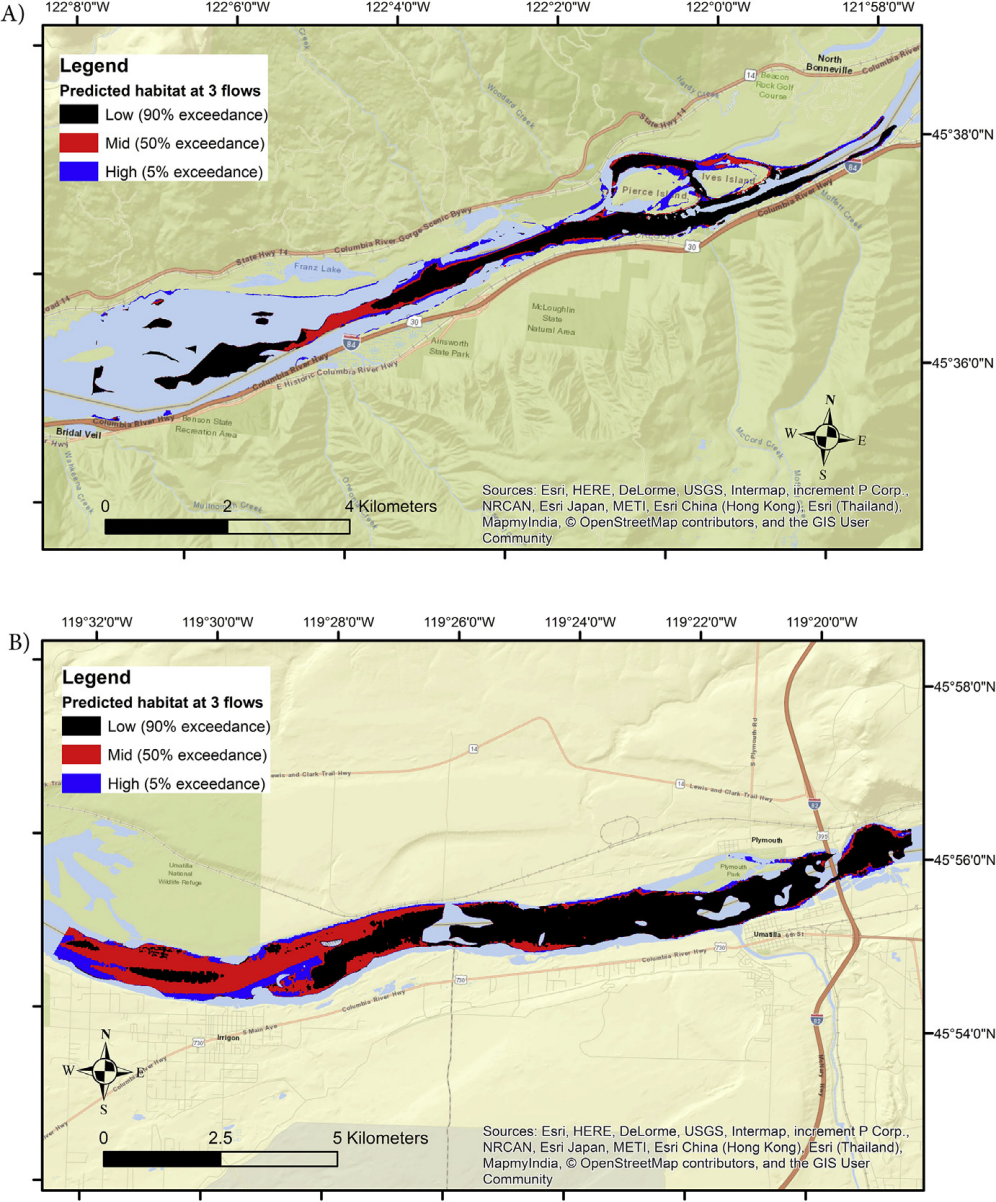
Binary habitat maps of Skamania (A – top panel) and John Day (B – bottom panel) reaches obtained from a Mahalanobis distance model at three flows. Two standard deviations (SD) were used as the binary threshold (SD ≤ 2 = predicted habitat; SD > 2 = unsuitable).

**Fig. 7:**
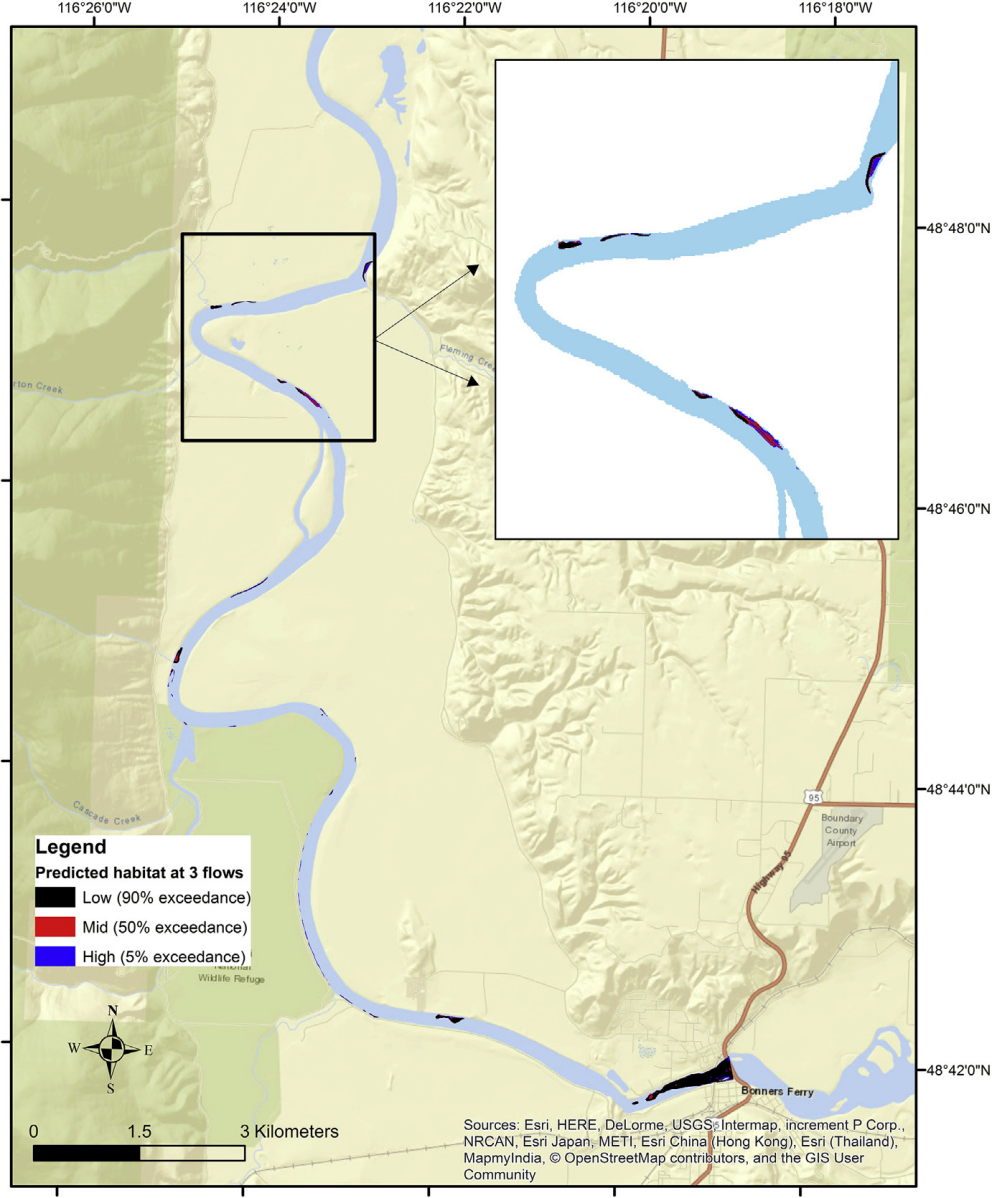
Predicted white sturgeon spawning habitat calculated at 3 flows in the Kootenai reach using a Mahalanobis distance model. Two standard deviations (SD) were used as the binary threshold (SD <= 2 = predicted habitat; SD > 2 = unsuitable). The map inset provides an enlargement of a portion of the reach where predicted White Sturgeon habitat occurred. A 5% exceedance flow produced the most predicted habitat (blue) and was always present underneath predicted habitat at 50% (red) or 90% (black) exceedance flows.

Binary habitat maps of three study reaches following application of Mahalanobis threshold (*D*^2^ ≤ 2 = habitat; *D*^2^ > 2 = unsuitable) revealed unique spatial patterns in predicted habitat. The Skamania (Fig. 6A) and John Day (Fig. 6B) reaches had considerable amounts of predicted spawning habitat throughout the reach, while Kootenai had most of its predicted habitat located near the head of the reach, with sparse amounts downstream in river bends (Fig. 7). Model probabilities at a given flow were correlated (*P* < 0.01) using Spearman's rank correlation (rho) test, while Wilcoxon test found median probabilities were different (*P* < 0.01). In each reach the highest ranked spawning habitat tended to be in upstream portions of the reach or in the thalweg, while lower model probabilities occurred downstream. The Kootenai reach contrasted with the other two reaches in that it had little predicted habitat throughout most of its reach. Detailed maps depicting Froude number, Mahalanobis distance, Mahalanobis Chi-square p-values, logistic probabilities, and binary habitat suitability maps are presented in Appendix 3 (Model outputs) for each reach.

**Fig. 8:**
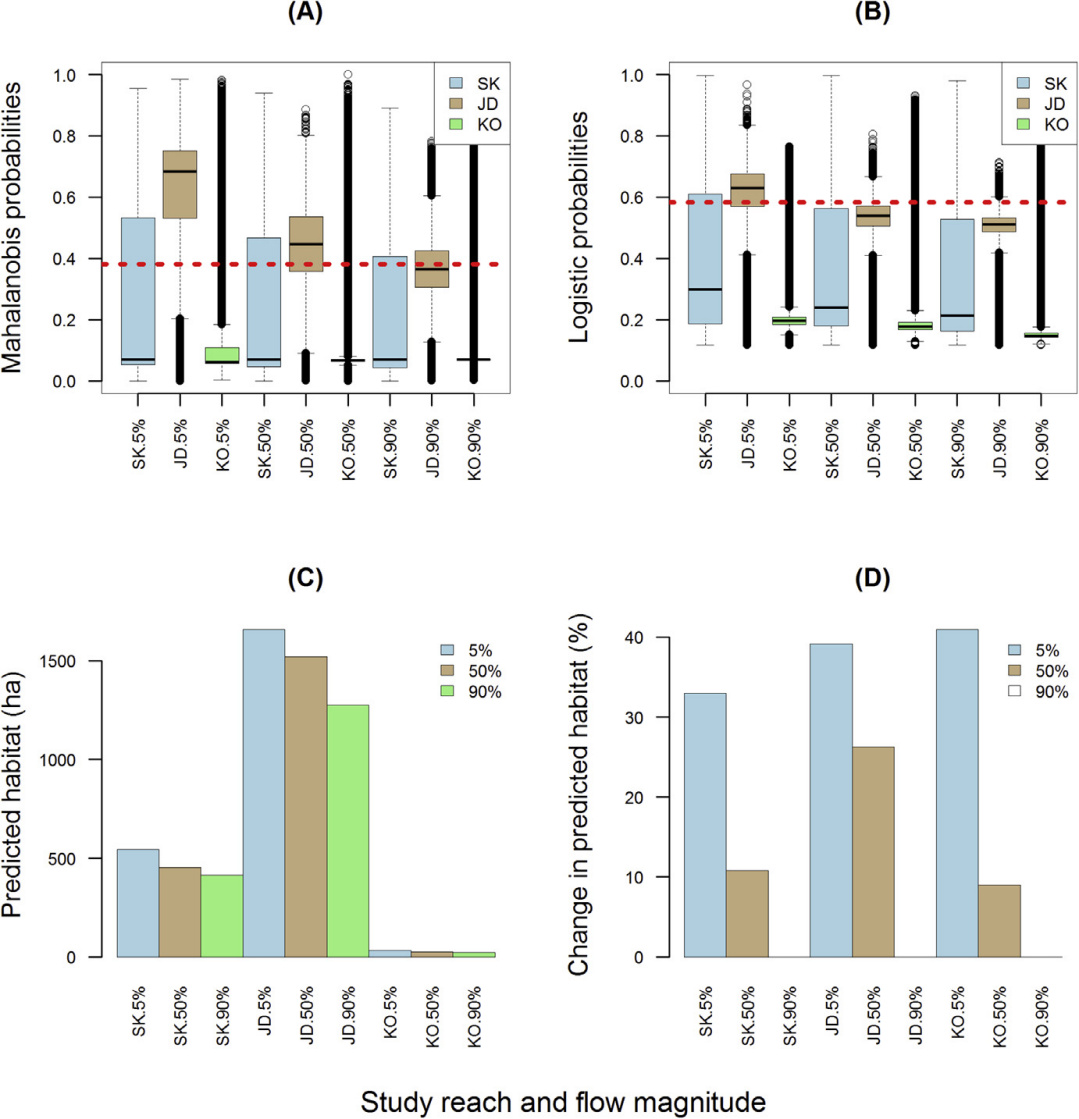
Boxplots of model probabilities for three reaches at three flows produced with Mahalanobis distance (A) and logistic regression (B) habitat models; amount of predicted sturgeon spawning habitat produced from both models (averaged) at three flows in three reaches (C), and predicted changes in habitat when flows increased from low to high, or low to moderate (D). Horizontal dashed abline in panels A and B represent the median model probability inside Skamania reach's training zone at a 5% exceedance flow (considered optimal). Boxplots display the 25th and 75th percentiles (bottom and top of box), medians (interior horizontal line), 5th and 95th percentiles (bottom and top lines outside box), and outliers (dots).

The overall pattern was similar between the Mahalanobis (equation (3)) and logistic (equation (4)) habitat models in that higher flows produced higher model probabilities in each reach (Fig. 8A,B). Boxplots revealed Skamania reach had the greatest variation in predicted habitat, indicating a more heterogeneous environment than the other reaches. John Day reach had the highest model probabilities and most predicted habitat (Fig. 8A,B) while Kootenai reach had the lowest model probabilities, smallest range, and least predicted habitat, with Skamania reach intermediate. The pattern of habitat formation relative to flow magnitude was similar between Mahalanobis and logistic models in Skamania and Kootenai reaches, therefore we averaged habitat estimates (Fig. 8C), with John Day producing the most predicted habitat followed in descending order by Skamania and Kootenai reaches. After we standardized habitat predictions by flow magnitude (subtracted 90% exceedance flow habitat estimate from 50% and 5% estimates), Kootenai reach produced the largest increase in predicted habitat when flow increased from low to high, followed by John Day and Skamania reaches, respectively (Fig. 8D). When flows went from low to medium, John Day reach produced the largest increase in predicted habitat, followed by Skamania and Kootenai reaches, respectively. Readers can interactively view and query online output from the Mahalanobis model at 3 flows in 3 reaches (see interactive maps). The naming conventions of the kmz files are as follows: reach_flow_model_habitat, thus the file named jd05_D2hab.kmz is predicted White Sturgeon habitat in John Day reach at a high (5% exceedance) flow, as determined from the Mahalanobis (D2) model, while sk90_D2hab.kmz is predicted White Sturgeon habitat in Skamania reach at a low (90% exceedance) flow using the Mahalanobis (D2) model. In all nine online maps, predicted White Sturgeon habitat is rendered in blue while unsuitable areas are rendered as yellow.

### 3.4 Model verification

**Fig. 9:**
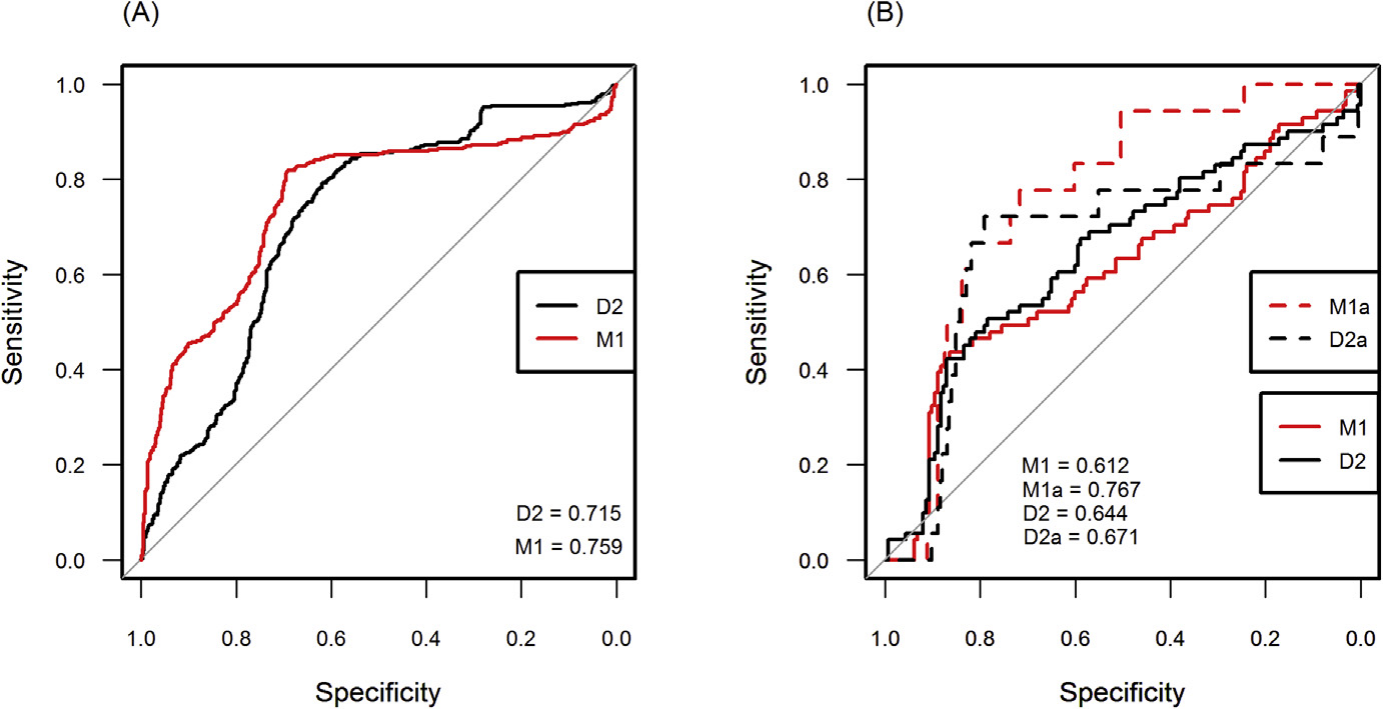
Classification accuracies achieved by Mahalanobis (D2) and logistic (M1) models inside Skamania reach (*n* = 969) with a Receiver Operating Characteristic curve (A), and classification accuracies achieved by each model following projection to Kootenai reach (*n* = 245) at two different spawning-per-unit-effort (SPUE) thresholds (B); M1 and D2 ROC calculated at 1 SPUE (71 presences/163 absences); M1a and D2a ROC calculated at 15 SPUE (18 presences/216 absences).

Areas under the ROC curve produced by a 20% set aside (*n* = 969) revealed the logistic model achieved a better overall classification result (AUC = 0.759) than Mahalanobis model (0.715) in Skamania reach (Fig. 9A). Specifically, the logistic model achieved higher classification success up until around 85% and then the curves crossed and Mahalanobis performed better. The logistic model at an 80% sensitivity threshold produced approximately 30% commission error (false positives) while Mahalanobis model produced approximately 40%, indicating pseudo-absence data aided the logistic model when defining a White Sturgeon spawning niche.

**Fig. 10:**
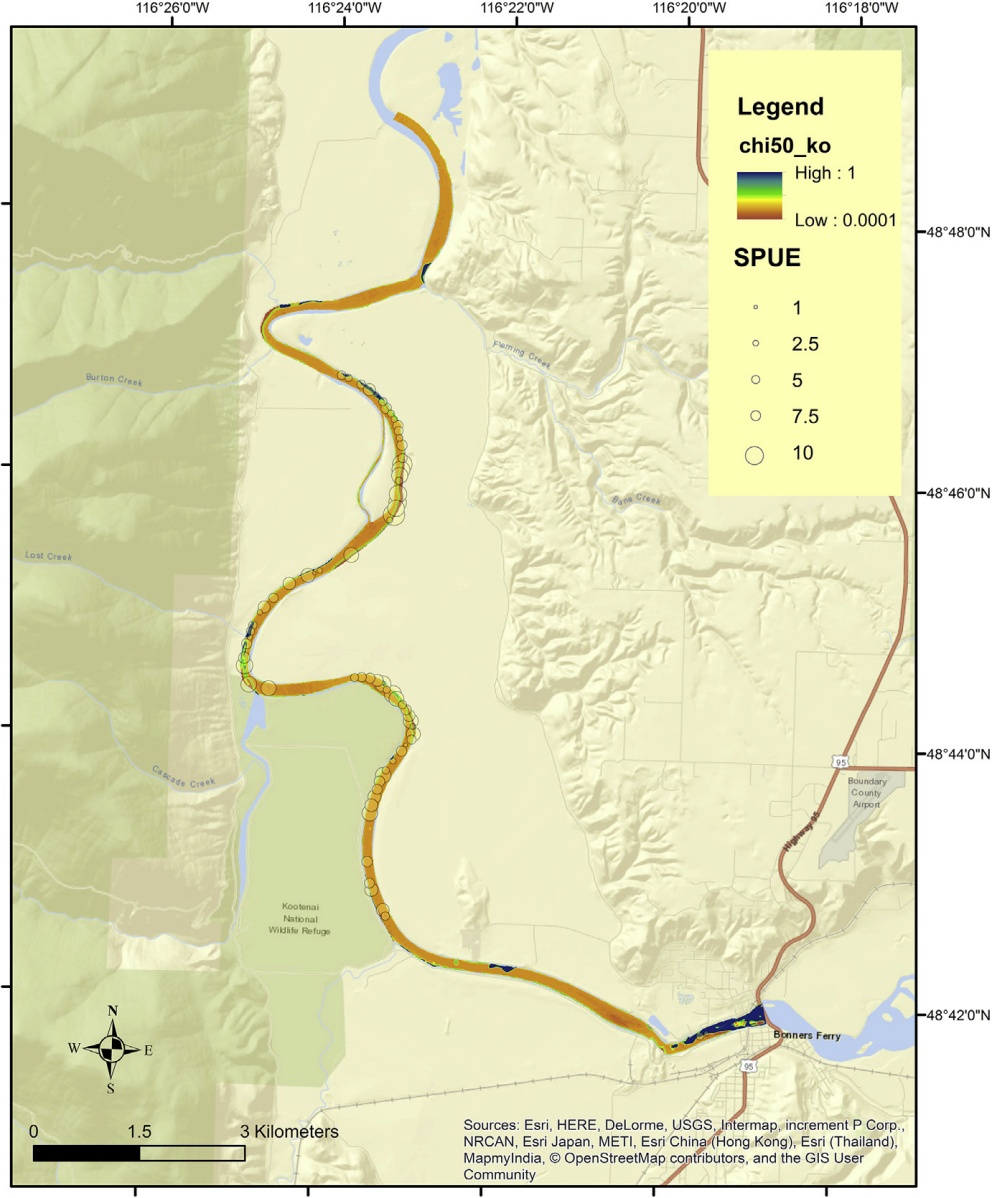
A proxy for White Sturgeon spawning habitat overlaid on Mahalanobis χ2 p-values (chi50_ko) in the Kootenai reach at a 50% exceedance flow - locations that had more White Sturgeon spawning (SPUE) are depicted with larger circles.

Following projection of habitat models into Kootenai reach and overlaying 234 spawning locations, areas under the ROC curves revealed Mahalanobis model achieved slightly higher classification results than the logistic model (0.644 and 0.612), respectively (Fig. 9B). While these classification results were not very robust they were significantly better than a random classifier (*P* < 0.05). Classification performance improved for each model when we shifted the SPUE detection threshold from 1 event to 15 events, with logistic model achieving an overall classification accuracy of 0.767 and Mahalanobis model 0.671 (Fig. 10). The change in appearance of ROC plots was reflective of the number of presences obtained at two different SPUE thresholds; SPUE ≥1 contained 71 spawning locations while SPUE ≥15 contained 18 locations.

### 3.5 Model sensitivity

**Fig. 11:**
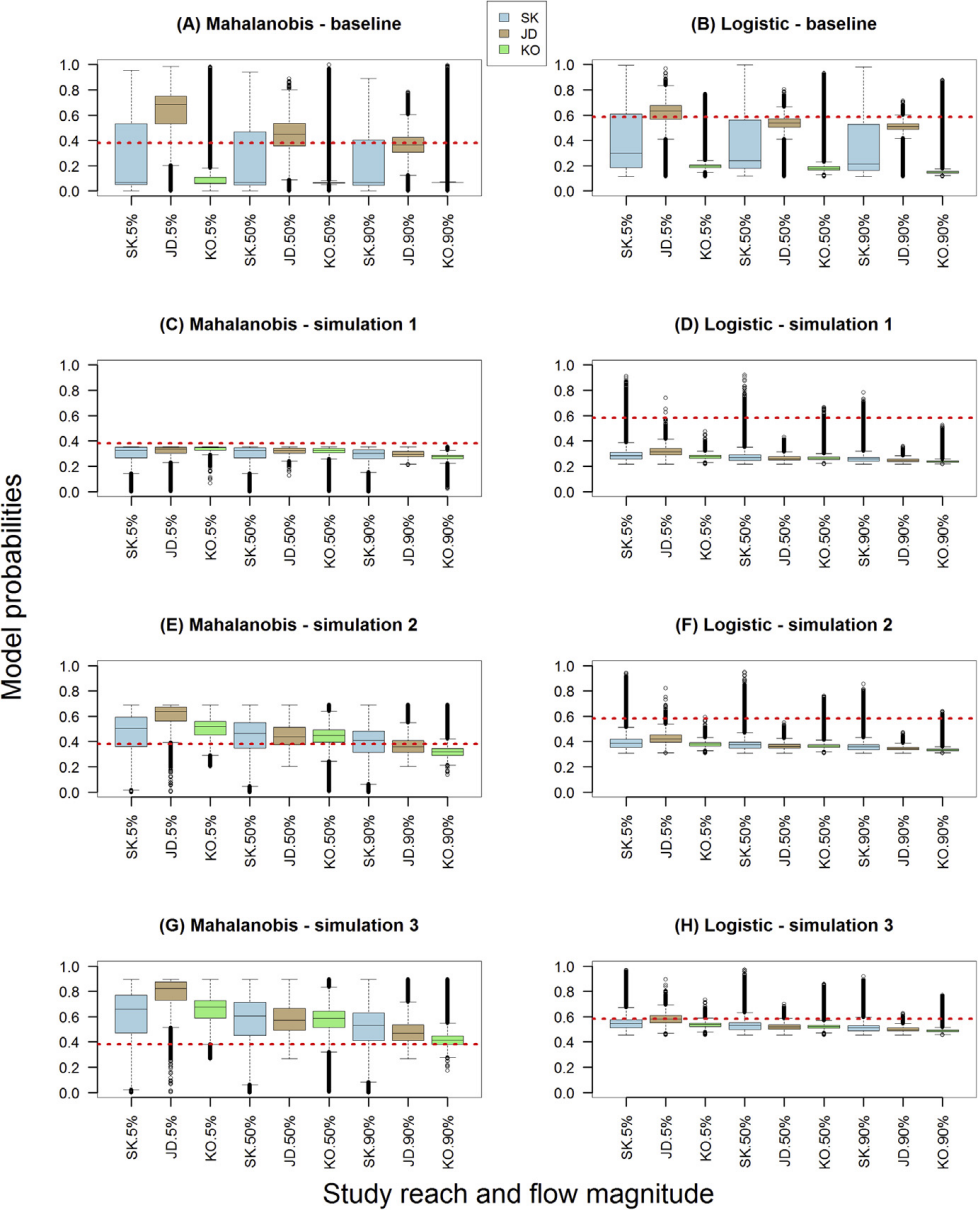
Model probabilities output by Mahalanobis and logistic models under contemporary conditions (A,B) and three simulations (C–H) whereby we globally changed gravel/cobble composition and embeddedness levels and recomputed model probabilities: simulation 1 (gravel/cobble = 10% & embeddedness = 50%); simulation 2 (gravel/cobble = 50% & embeddedness = 10%); simulation 3 (gravel/cobble = 90% & embeddedness = 5%). Horizontal abline (dashed line) equals the median model probability observed in Skamania reach's training zone at a 5% exceedance flow (considered optimal). Boxplots display the 25th and 75th percentiles (bottom and top of box), medians (interior horizontal line), 5th and 95th percentiles (bottom and top lines outside box), and outliers (dots).

Changing substrate and embeddedness values globally and recomputing Mahalanobis and logistic model probabilities produced noticeable changes when compared to baseline (contemporary) conditions in three reaches at three flows (Fig 11A,B). Simulation one - where we globally changed gravel/cobble composition to 10% and embeddedness to 50% – produced a positive improvement in Kootenai reach model probabilities (Fig. 11C,D). Results were opposite in John Day reach where model probabilities moved downwards and resulted in a truncation in range. Median model probabilities increased in Skamania reach but the upper quartiles were all below the abline due to a truncation in range. The overall pattern improved (model probabilities increased) in simulation 2 after we globally increased cobble/gravel composition to 50% and decreased embeddedness values to 10% (Fig. 11E,F). Both models' overall patterns were similar although the Mahalanobis model responded more to global changes than the logistic model, moving higher up the probability scale. In simulation three – where we increased gravel/cobble composition to 90% and reduced embeddedness to 5% – all model outputs improved, with Mahalanobis median probabilities well above the abline and logistic outputs either at or just below the abline (Fig. 11G,H).

## 4 Discussion

### 4.1 Habitat response to flow by reach

The distinguishing feature of Skamania reach compared to John Day and Kootenai reaches was its diversity in hydraulics, channel morphology, and substrate characteristics. Driving this diversity were islands, side channels, and tributary junctions. Another key difference was Skamania reach is not backwatered (although it does have a minor tidal influence) by downstream dams, explaining why it achieved the smallest gains in predicted habitat as a response to flow magnitude (i.e., it already produces habitat at all flows). All of these conditions collectively make Skamania reach a very diverse and dynamic area with a robust, healthy White Sturgeon population. While diversity in channel morphology and hydraulics were not predictors in our analysis due to a small sample size (three reaches), they are thought to be important habitat features rangewide (Hildebrand et al., 2016). The diversity of physical conditions observed in Skamania reach revealed that a successful White sturgeon spawning reach can contain areas with fine sediments and high embeddedness provided there are areas with swift water and gravel/cobble substrates.

The John Day reach produced more predicted White Sturgeon habitat than the other two reaches due to its excellent gravel/cobble substrate and very low embeddedness levels, yet it experiences intermittent White Sturgeon recruitment, demonstrating that substrate characteristics can't be solely responsible. Our simulations revealed that predicted habitat increased more in John Day reach compared to Skamania reach when flows increased from low to high, likely due to the backwatering effect from downstream John Day Dam that increased surface area and reduced Froude values at lower flows. The positive relation between flow magnitude and predicted White Sturgeon spawning habitat has been documented previously (Parsley and Beckman, 1994), and the relation between flow magnitude and recruitment continues to be documented in this reach (Rybacki et al., 2017). Thus, the key appears to be the combination of physical factors identified in our habitat models (substrate, embeddedness, flow magnitude), versus one factor or another. Flow magnitude has been suppressed in all reaches since completion of upriver storage reservoirs, and our simulations provide more evidence that flow optimization at McNary Dam - or reducing the backwater effect by lowering the pool level of John Day Reservoir during the White Sturgeon spawning period – could improve White Sturgeon recruitment in this reach (Parsley and Beckman, 1994).

Kootenai reach had the smallest range of model probabilities, indicating a lack of channel diversity compared to John Day or Skamania reaches. Our simulations revealed that larger spring flows in Kootenai reach increased predicted White Sturgeon spawning habitat without altering substrate characteristics, but only when we altered substrate composition and embeddedness levels to more closely match Skamania reach did model probabilities resemble Skamania and John Day reaches. The current physical conditions in Kootenai reach changed significantly from those observed under pre-dam conditions before Libby Dam became operational in 1974 (USFWS, 1994, 2008). Specifically, hydropower operations and flood control activities reduced the magnitude of spring flows while diking and channelization altered sediment transport, resulting in a complete failure of white sturgeon recruitment for four decades (USFWS, 2008). Given that flushing flows sweep away fine sediments and expose buried gravels and cobbles (Barton et al., 2005; McDonald et al., 2010), flow management combined with channel alterations will be necessary to restore optimal spawning conditions in Kootenai reach (Paragamian and Rust, 2014).

### 4.2 Habitat restoration and conservation aquaculture

Within the Kootenai (Meander) reach, and further upstream (Braided reach), there are active channel restoration and enhancement activities to see if white sturgeon recruitment failure can be overcome. Specifically, the Kootenai River Habitat Restoration Program is an ongoing ecosystem-based river habitat restoration effort spanning an 88-km reach of the Kootenai River in north Idaho (KTOI, 2009; KTOI, 2018). Pool forming structures, substrate enhancement, channel alterations, and riparian restoration activities are being conducted since 2011 in strategic locations to encourage white sturgeon to migrate and spawn in restored/enhanced habitats. In addition, experimental high flows are being conducted in the spring to flush sediments and cue white sturgeon spawning. The channel restoration/enhancement approach is an attempt to work within the flood control constraints of the FCRPS given that the high spring flows observed before Libby Dam would likely cause considerable property damage to downstream residents. White sturgeon recruitment has not been observed in Kootenai reach but it is hoped that these activities will produce the right combination of factors that cue white sturgeon to spawn successfully in suitable substrates without serious alterations to Columbia River system operations.

Conservation aquaculture is being used in conjunction with flow optimization and habitat restoration in the Kootenai River to overcome white sturgeon recruitment failure (KTOI, 2012). Specifically, wild sturgeon broodstock are collected for eggs and sperm each year and offspring are raised to 1-year-olds in a hatchery before release into the wild. Hundreds of thousands of white sturgeon juveniles have been released into the Kootenai River and about 10% have survived. Now, many hatchery-reared white sturgeons are approaching breeding age (∼16-years-old) and will begin spawning in the Kootenai River in upcoming years. No one knows if the offspring of these fish will overcome the recruitment bottleneck, but hopes are high that their sheer numbers in conjunction with the extensive channel modifications and experimental flow pulses will result in some white sturgeon successfully spawning once again in the Kootenai River.

## 5 Conclusion

Our habitat models revealed that Kootenai reach is the most responsive to flow magnitude and is very sensitive to global environmental changes, suggesting habitat restoration and enhancement activities could produce significant gains in this reach. Given the endangered status of White Sturgeon in the Kootenai reach (USFWS, 2008), our spatially explicit models could provide guidance to natural resource agencies in their efforts to restore and enhance this reach or other locations in the basin that have White Sturgeon stocks. Specifically, our models could be used in the development of a decision support system (DSS) to guide restoration/enhancement activities and to evaluate their effectiveness. For example, our sensitivity analysis focused on global changes to a reach, but they could also be used to simulate changes to specific areas within a reach or to evaluate effectiveness of management actions in a spatially explicit manner. Evaluating changes in a reach will require that our habitat models be populated with new bathymetry, substrate, and hydraulic data wherever a management action (e.g., restoration) occurs so that model probabilities can be recomputed and compared to prior model runs. An alternative would be to simulate changes prior to implementing changes by artificially altering the database in such a way as to mimic proposed changes (see Fig. 11), but in specific locations rather than globally and recomputing model probabilities. Given that the location of every cell was mapped in 2008, the transition to a spatially explicit DSS is realistic and achievable provided new data are available. Our models also provide a foundation upon which a rangewide white sturgeon meta-analysis could be conducted to examine underlying causes of recruitment success and failure using standardized metrics.

## Declarations

### Author contribution statement

James Hatten, Michael Parsley, Gary Barton: Conceived and designed the experiments; Performed the experiments; Analyzed and interpreted the data; Contributed reagents, materials, analysis tools or data; Wrote the paper.

Ryan Fosness, Thomas Batt: Performed the experiments; Analyzed and interpreted the data; Wrote the paper.

### Funding statement

The Bonneville Power Administration funded this work under contracts 28424, 35990, and 40084 as part of the larger Kootenai Tribe of Idaho's Project 2002-00-200. Final analysis and manuscript preparation were completed with U.S. Geological Survey Priority Landscapes project dollars.

### Competing interest statement

The authors declare no conflict of interest.

### Additional information

Supplementary content related to this article has been published online at https://doi.org/10.1016/j.heliyon.2018.e00629.

Interactive map data associated with this study is available online at http://www.sciencedirect.com/science/article/pii/S2405-8440(17)33282-6.

## Appendix A Supplementary data

Appendix 1. Mapping substrate and embeddedness Appendix 1. Mapping substrate and embeddedness

Appendix 2. Substrate Froude and embeddedness maps Appendix 2. Substrate Froude and embeddedness maps

Appendix 3. Model outputs Appendix 3. Model outputs

jd05_D2hab.kmz jd05_D2hab.kmz is predicted White Sturgeon habitat in John Day reach at a high (5% exceedance) flow, as determined from the Mahalanobis (D2) model. Predicted White Sturgeon habitat is rendered in blue while unsuitable areas are rendered as yellow. jd05_D2hab.kmz

jd50_D2hab.kmz jd50_D2hab.kmz is predicted White Sturgeon habitat in John Day reach at a medium (50% exceedance) flow, as determined from the Mahalanobis (D2) model. Predicted White Sturgeon habitat is rendered in blue while unsuitable areas are rendered as yellow. jd50_D2hab.kmz

jd90_D2hab.kmz jd90_D2hab.kmz is predicted White Sturgeon habitat in John Day reach at a low (90% exceedance) flow, as determined from the Mahalanobis (D2) model. Predicted White Sturgeon habitat is rendered in blue while unsuitable areas are rendered as yellow. jd90_D2hab.kmz

ko05_D2hab.kmz ko05_D2hab.kmz is predicted White Sturgeon habitat in Kootenai (Meander) reach at a high (5% exceedance) flow, as determined from the Mahalanobis (D2) model. Predicted White Sturgeon habitat is rendered in blue while unsuitable areas are rendered as yellow. ko05_D2hab.kmz

ko50_D2hab.kmz ko50_D2hab.kmz is predicted White Sturgeon habitat in Kootenai (Meander) reach at a medium (50% exceedance) flow, as determined from the Mahalanobis (D2) model. Predicted White Sturgeon habitat is rendered in blue while unsuitable areas are rendered as yellow. ko50_D2hab.kmz

ko90_D2hab.kmz ko90_D2hab.kmz is predicted White Sturgeon habitat in Kootenai (Meander) reach at a low (90% exceedance) flow, as determined from the Mahalanobis (D2) model. Predicted White Sturgeon habitat is rendered in blue while unsuitable areas are rendered as yellow. ko90_D2hab.kmz

sk05_D2hab.kmz sk05_D2hab.kmz is predicted White Sturgeon habitat in Skamania reach at a high (5% exceedance) flow, as determined from the Mahalanobis (D2) model. Predicted White Sturgeon habitat is rendered in blue while unsuitable areas are rendered as yellow. sk05_D2hab.kmz

sk50_D2hab.kmz sk50_D2hab.kmz is predicted White Sturgeon habitat in Skamania reach at a medium (50% exceedance) flow, as determined from the Mahalanobis (D2) model. Predicted White Sturgeon habitat is rendered in blue while unsuitable areas are rendered as yellow. sk50_D2hab.kmz

sk90_D2hab.kmz sk90_D2hab.kmz is predicted White Sturgeon habitat in Skamania reach at a low (90% exceedance) flow, as determined from the Mahalanobis (D2) model. Predicted White Sturgeon habitat is rendered in blue while unsuitable areas are rendered as yellow. sk90_D2hab.kmz

## References

[bib1] Anders P.J., Richards D.L., Powell M.S., Van Winkle W., Anders P.J., Secor D.H., Dixon D. (2002). The first endangered white sturgeon population: repercussions in an altered large river-floodplain ecosystem. Biology, Management, and Protection of North American Sturgeon.

[bib2] Baker D.W., McAdam D.S.O., Boucher M., Huynh K.T., Brauner C.J. (2014). Swimming performance and larval quality are altered by rearing substrate at early life stages in white sturgeon, *Acipenser transmontanus* (Richardson, 1836). J. Appl. Ichthyol..

[bib3] Barton G.J., McDonald R.R., Nelson J.M., Dinehart R.L. (2005). Simulation of Flow and Sediment Mobility Using a Multidimensional Flow Model for the white sturgeon Critical-habitat Reach, Kootenai River near Bonners Ferry, Idaho: U.S. Geological Survey Scientific Investigations Report 2005-5230.

[bib4] Bevelhimer M.S. (2002). A bioenergetics model for white sturgeon *Acipenser transmontanus*: assessing differences in growth and reproduction among Snake River reaches. J. Appl. Ichthyol..

[bib5] Boucher M.A., McAdam S.O., Shrimpton J.M. (2014). The effect of temperature and substrate on the growth, development and survival of larval white sturgeon. Aquaculture.

[bib6] Burnham K.P., Anderson D.R. (2002). Model Selection and Multimodel Inference: a Practical Information—Theoretic Approach.

[bib7] Clark J.D., Dunn J.E., Smith K.G. (1993). A multivariate model of female black bear habitat use for a geographic information system. J. Wildl. Manag..

[bib8] Dauble D.D., Hanrahan T.P., Geist D.R., Parsley M.J. (2003). Impacts of the Columbia River hydroelectric system on main-stem habitats of fall Chinook salmon. N. Am. J. Fish. Manag..

[bib9] Egan J.P. (1975). Signal Detection Theory and ROC Analysis.

[bib10] Fish M. (2010). A white sturgeon year-class index for the San Francisco Estuary and its relation to delta outflow. IEP Newslett..

[bib11] Hatten J.R., Batt T.R. (2010). Hydraulic alterations resulting from hydropower development in the Bonneville reach of the Columbia River. Northwest Sci..

[bib12] Hatten J.R., Parsley M.J. (2009). A spatial model of white sturgeon rearing habitat in the lower Columbia River, USA. Ecol. Model..

[bib13] Hatten J.R., Tiffan K.F., Anglin D.R., Haeseker S.L., Skalicky J.J., Schaller H. (2009). A spatial model to assess the effects of hydropower operations on Columbia River fall Chinook salmon spawning habitat. N. Am. J. Fish. Manag..

[bib14] Hildebrand L.R., Schreier A.D., Lepla K., McAdam S.O., McLellan J., Parsley M.J., Paragamian V.L., Young S.P. (2016). Status of White Sturgeon (Acipenser transmontanus Richardson, 1863) throughout the species range, threats to survival, and prognosis for the future. J. Appl. Ichthyol..

[bib15] Hosmer D.W., Lemeshow S. (2000). Applied Logistic Regression.

[bib16] Jackson Z.J., Gruber J.J., Van Eenennaam J.P. (2016). White sturgeon spawning in the san Joaquin river, California, and effects of water management. J. Fish. Wildl. Manag..

[bib17] Johnson M.J., Hatten J.R., Holmes J.A., Shafroth P.B. (2017). Identifying western yellow-billed cuckoo breeding habitat with a dual modelling approach. Ecol. Model..

[bib18] Jowett I.G., Richardson J., Biggs B.J.F., Hickey C.W., Quinn J.M. (1991). Microhabitat preferences of benthic invertebrates and the development of generalized *Deleatidium* spp. Habitat suitability curves, applied to four New Zealand rivers. New Zeal. J. Mar. Fresh..

[bib19] Jowett I.G. (1993). A method for objectively identifying pool, run, and riffle habitats from physical measurements. New Zeal. J. Mar. Fresh..

[bib20] Koch T.J., Congleton J.L., Anders P.J. (2006). Effects of sediment cover on survival and development of white sturgeon embryos. N. Am. J. Fish. Manag..

[bib21] Kohlhorst D.W., Botsford L.W., Brennan J.S., Caillet G.M., Williot P. (1991). Aspects of the structure and dynamics of an exploited central California population of white sturgeon (*Acipenser transmontanus*). Acipenser. Actes du premier colloque international sur l'esturgeon.

[bib22] KTOI (Kootenai Tribe of Idaho) (2009). Kootenai River Habitat Restoration Project Master Plan: a Conceptual Feasibility Analysis and Design Framework.

[bib23] KTOI (Kootenai Tribe of Idaho) (2012). Technical Basis for the Kootenai Sturgeon Conservation Aquaculture Program.

[bib24] KTOI (Kootenai Tribe of Idaho) (2018). Kootenai River Habitat Restoration Program. http://www.restoringthekootenai.org/.

[bib25] Lamouroux N., Poff N.L., Angermeier P.L. (2002). Intercontinental convergence of stream fish community traits along geomorphic and hydraulic gradients. Ecology.

[bib26] Mahalanobis P.C. (1936). On the generalized distance in statistics. Proc. Nat. Inst. Sci. Ind..

[bib27] McAdam S.O. (2011). Effects of substrate condition on habitat use and survival by white sturgeon (*Acipenser transmontanus*) larvae, and potential implications for recruitment. Can. J. Fish. Aquat. Sci..

[bib28] McAdam D.S.O. (2012). Diagnosing white sturgeon (*Acipenser transmontanus*) Recruitment Failure and the Importance of Substrate Condition to Yolksac Larvae Survival.

[bib29] McAdam D.S.O. (2015). Retrospective weight-of-evidence analysis identifies substrate change as the apparent cause of recruitment failure in the upper Columbia River white sturgeon (*Acipenser transmontanus*). Can. J. Fish. Aquat. Sci..

[bib30] McAdam S.O., Walters C.J., Nistor C. (2005). Linkages between white sturgeon recruitment and altered bed substrates in the Nechako River, Canada. Trans. Am. Fish. Soc..

[bib31] McDonald R., Nelson J., Paragamian V., Barton G. (2010). Modeling the effect of flow and sediment transport on white sturgeon spawning habitat in the Kootenai River, Idaho. J. Hydraul. Eng..

[bib32] Miller A.I., Counihan T.D., Parsley M.J., Beckman L.G., LaRoe E.T., Farris G.S., Puckett C.E., Doran P.D., Mac M.J. (1995). Columbia River basin white sturgeon. Our Living Resources: a Report to the Nation on the Distribution, Abundance, and Health of U.S. Plants, Animals, and Ecosystems.

[bib33] Mora E.A., Lindley S.T., Erickson D.L., Klimley A.P. (2009). Do impassable dams and flow regulation constrain the distribution of green sturgeon in the Sacramento River, California?. J. Appl. Ichthyol..

[bib34] Paragamian V.L., Rust P. (2014). Validation of a methodology to determine female white sturgeon (*Acipenser transmontanus* Richardson, 1836) habitat use within a riverscape during the spawning season. J. Appl. Ichthyol..

[bib35] Paragamian V.L., McDonald R., Nelson G.J., Barton G. (2009). Kootenai River velocities, depth, and white sturgeon spawning site selection: a mystery unraveled?. J. Appl. Ichthyol..

[bib36] Parsley M.J., Anders P.J., Miller A.I., Beckman L.G., McCabe G.T., Webster Van Winkle P.A., Secor David H., Dixon Doug (2002). Recovery of white sturgeon populations through natural production: understanding the influence of abiotic and biotic factors on spawning and subsequent recruitment.

[bib37] Parsley M., Beckman L., McCabe G. (1993). Spawning and rearing habitat use by white sturgeons in the Columbia River downstream from McNary Dam. T. Am. Fish. Soc..

[bib38] Parsley M.J., Beckman L.G. (1994). White sturgeon spawning and rearing habitat in the Lower Columbia River. N. Am. J. Fish. Manag..

[bib39] Parsley M.J., Wright C.D., van der Leeuw B.K., Kofoot E.E., Peery C.A., Moser M.L. (2007). White Sturgeon (*Acipenser transmontanus*) passage at the Dalles dam, Columbia River, USA. J. Appl. Ichthyol..

[bib40] Perrin C.J., Rempel L.L., Rosenau M.L. (2003). White sturgeon spawning habitat in an unregulated river: Fraser River, Canada. T. Am. Fish. Soc..

[bib41] R Core Team (2017). R: a Language and Environment for Statistical Computing. https://www.R-project.org/.

[bib42] Robin X., Turck N., Hainard A. (2011). pROC: an open-source package for R and S+ to analyze and compare ROC curves. BMC Bioinf..

[bib43] Rybacki K.J., Stevens P.M., Chapman C.G. (2017). White sturgeon Mitigation and Restoration in the Columbia and Snake Rivers Upstream from Bonneville Dam: Evaluate the success of Developing and Implementing a Management Plan to Enhance Production of White Sturgeon in Reservoirs between Bonneville and McNary Dams.

[bib44] Schaffter R.G. (1997). White sturgeon spawning migrations and location of spawning habitat in the Sacramento River, California. Calif. Fish Game.

[bib45] Stevens D.E., Miller L.W. (1970). Distribution of sturgeon larvae in the Sacramento-San Joaquin river system. Calif. Fish Game.

[bib46] Tiffan K.F., Garland R.D., Rondorf D.W. (2002). Quantifying flow-dependent changes in subyearling fall chinook salmon rearing habitat using two-dimensional spatially explicit modeling. N. Am. J. Fish. Manag..

[bib47] Tukey J.W. (1977). Exploratory Data Analysis.

[bib48] U.S. Fish and Wildlife Service (USFWS) (1994). Endangered and threatened wildlife and plants; determination of endangered status for the Kootenai River population of white sturgeon—final rule. Fed. Regist..

[bib49] U.S. Fish and Wildlife Service (USFWS) (2008). Endangered and threatened wildlife and plants; critical habitat revised designation for the Kootenai river population of the white sturgeon (*Acipenser transmontanus*); final rule. Fed. Regist..

[bib50] van der Leeuw B.K., Parsley M.J., Wright C.D., Kofoot E.E. (2006). Validation of a Critical assumption of the Riparian Habitat Hypothesis for white sturgeon: U.S. Geological Survey Scientific Investigations Report 2006-5225.

