# Supplementary material for: Substrate and flow characteristics associated with White Sturgeon recruitment in the Columbia River Basin
Source: Heliyon. 2018 May 21;4(5):e00629. doi: 10.1016/j.heliyon.2018.e00629 (PMC5986543; doi:10.1016/j.heliyon.2018.e00629)
Supplement: Appendix 1. Mapping substrate and embeddedness [file mmc1.docx]

**Appendix 1:** Mapping Substrate Composition and Embeddedness in the Skamania, John Day, and Kootenai Reaches

**A1.1. Introduction**

A primary goal of this project is the comparison of physical features found in river reaches that have consistent (Skamania), intermittent (John Day), or negligible (Kootenai) White Sturgeon recruitment. In this chapter we describe the equipment and methodology used to collect and analyze substrate composition and embeddedness, and we characterize and contrast the three study reaches. Prior substrate maps and data existed for each reach, but the data were too coarse to allow habitat assessments to be derived, so we created new substrate composition and embeddedness maps. This information was necessary as inputs into the 2D hydrodynamic models and for the White Sturgeon spawning habitat models. Bottom roughness is a primary input parameter when calibrating a 2D hydro model, while substrate composition and embeddedness are important features and conditions in the assessment of White Sturgeon spawning habitat. The substrate and embeddedness maps also provide a baseline for future physical or habitat comparisons should the need arise.

**A1.2. Methods**

*A1.2.1. Video samples*

We collected video samples throughout the three study reaches to characterize substrate composition and embeddedness. We placed an emphasis on areas that transitioned between morphological features, such as between the thalweg and shallow areas, or between riffles and glides. We used a Global Positioning System (GPS) and GIS to create navigation tracks for substrate sampling in the Skamania (Fig. A1.1A), John Day (Fig. A1.1B), and Kootenai (Fig. A1.2) reaches. We created a customized substrate grab-sampler to house a SeaViewer color-video camera and video lights (Fig. A1.3). The grab-sampler frame had a 17.7-cm viewer that framed each video sample, allowing the substrate to be scaled to a common reference. We first

Figure A1.1. Locations of video samples used for substrate composition and embeddedness mapping in the Skamania (A) and John Day (B) reaches.

A)


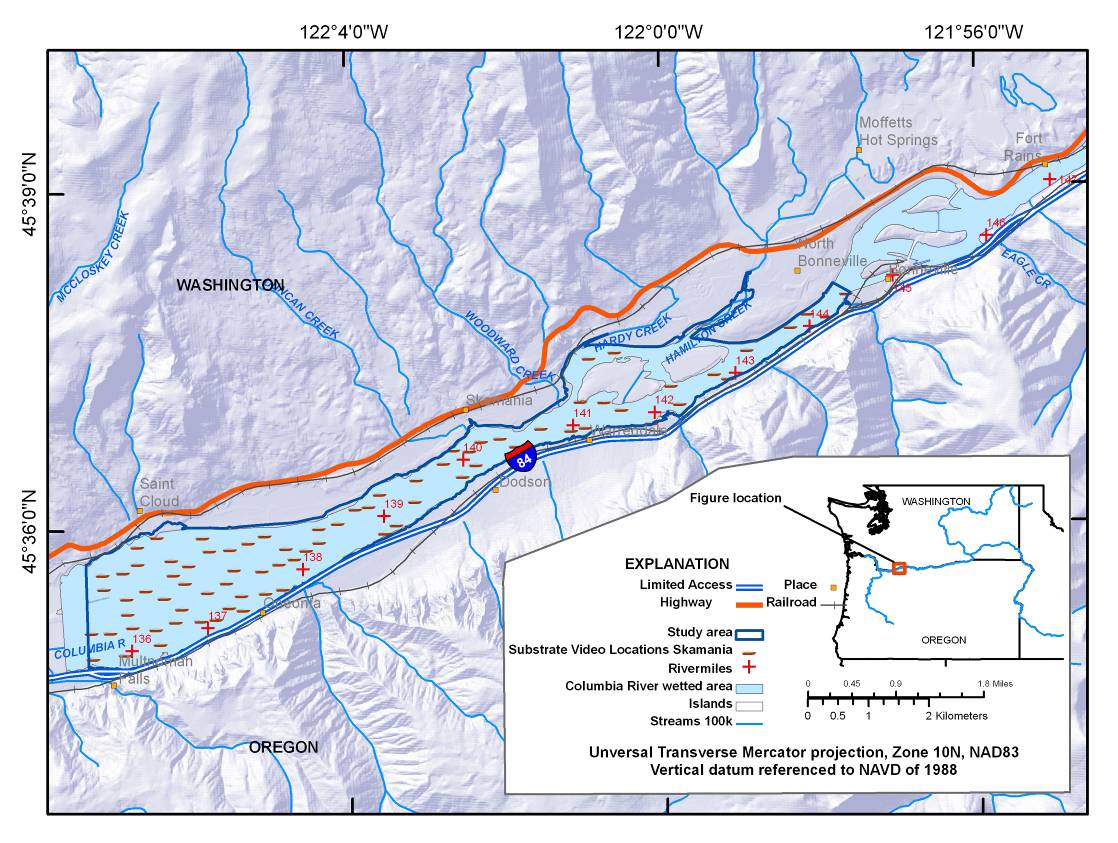

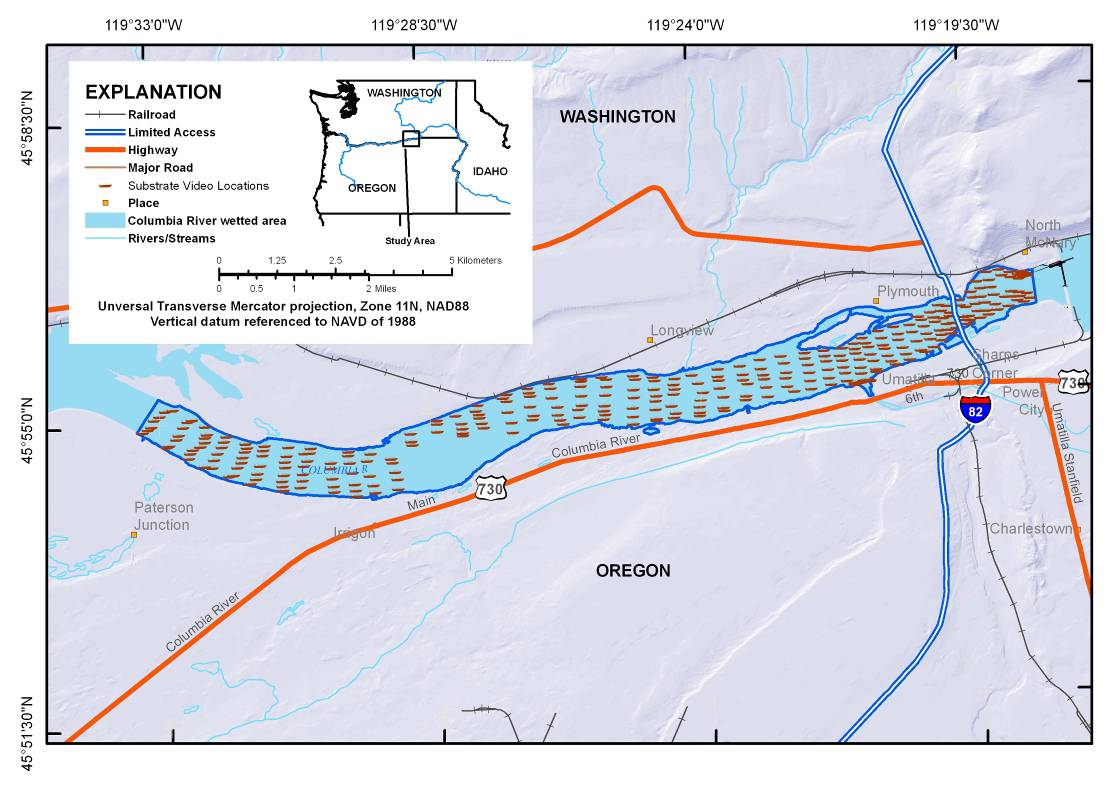


B)

activated the video camera and then lowered it to the river bottom, allowing us to capture the sediment plume resulting from the sampler when it first contacted the bottom. Images of the sediment plume, plus a sharp image of the river bottom after the sediment plume cleared, gave us considerable information about substrate composition and embeddedness. We collected a total of 255 video samples in 2008, plus we utilized 2000 video and visual samples around Ives Island (Skamania reach) that were collected in a prior study (Garland et al. 2003). In the John Day reach, we collected 302 video samples in 2008 using our modified grab-sampler frame. In the Kootenai reach, we collected 1,265 substrate samples. When possible, we examined video files on site to determine if another video sample was necessary before moving to the next site, but wind and currents sometimes prevented repeat samples.

Figure A1.2. Locations of video samples used for substrate composition and embeddedness mapping in the Kootenai reach.


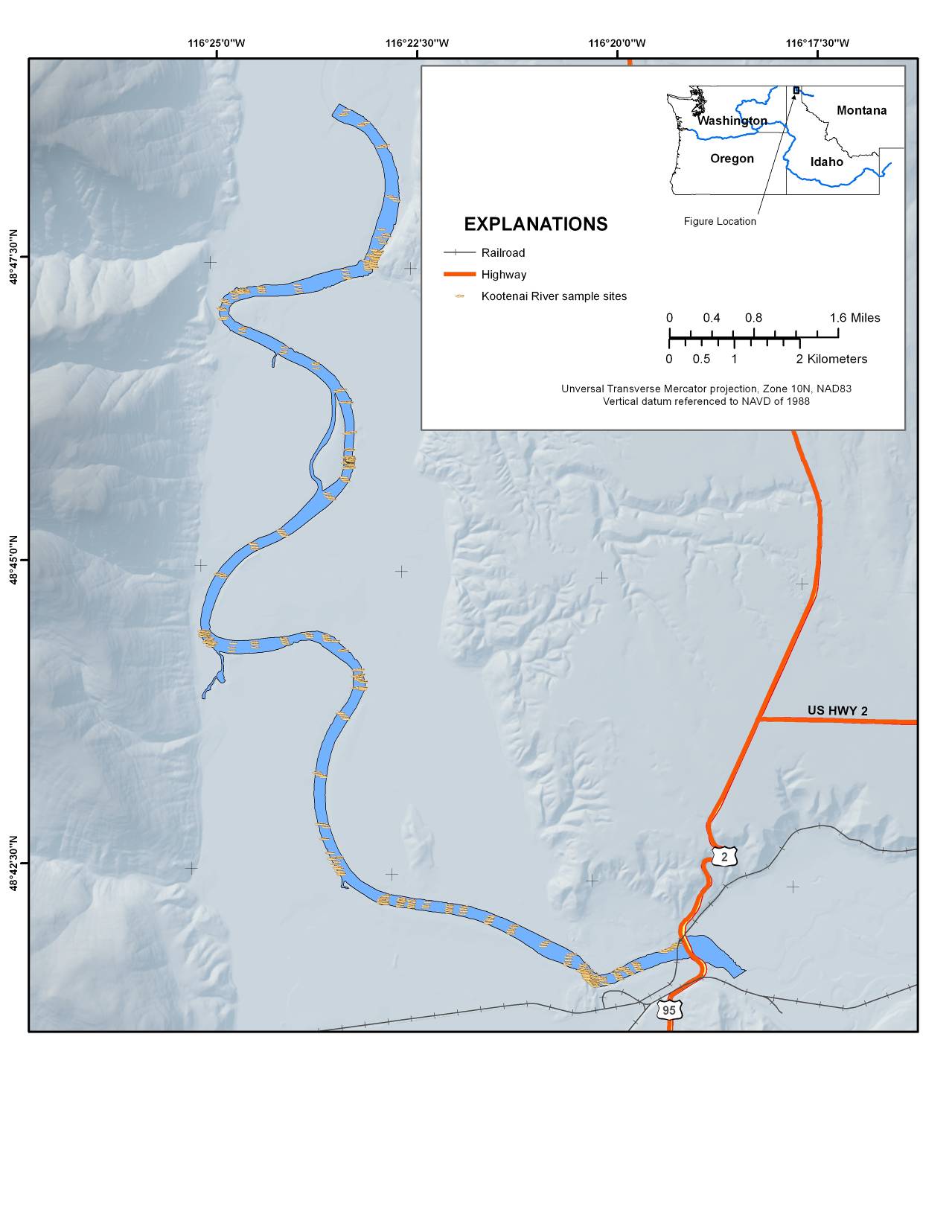

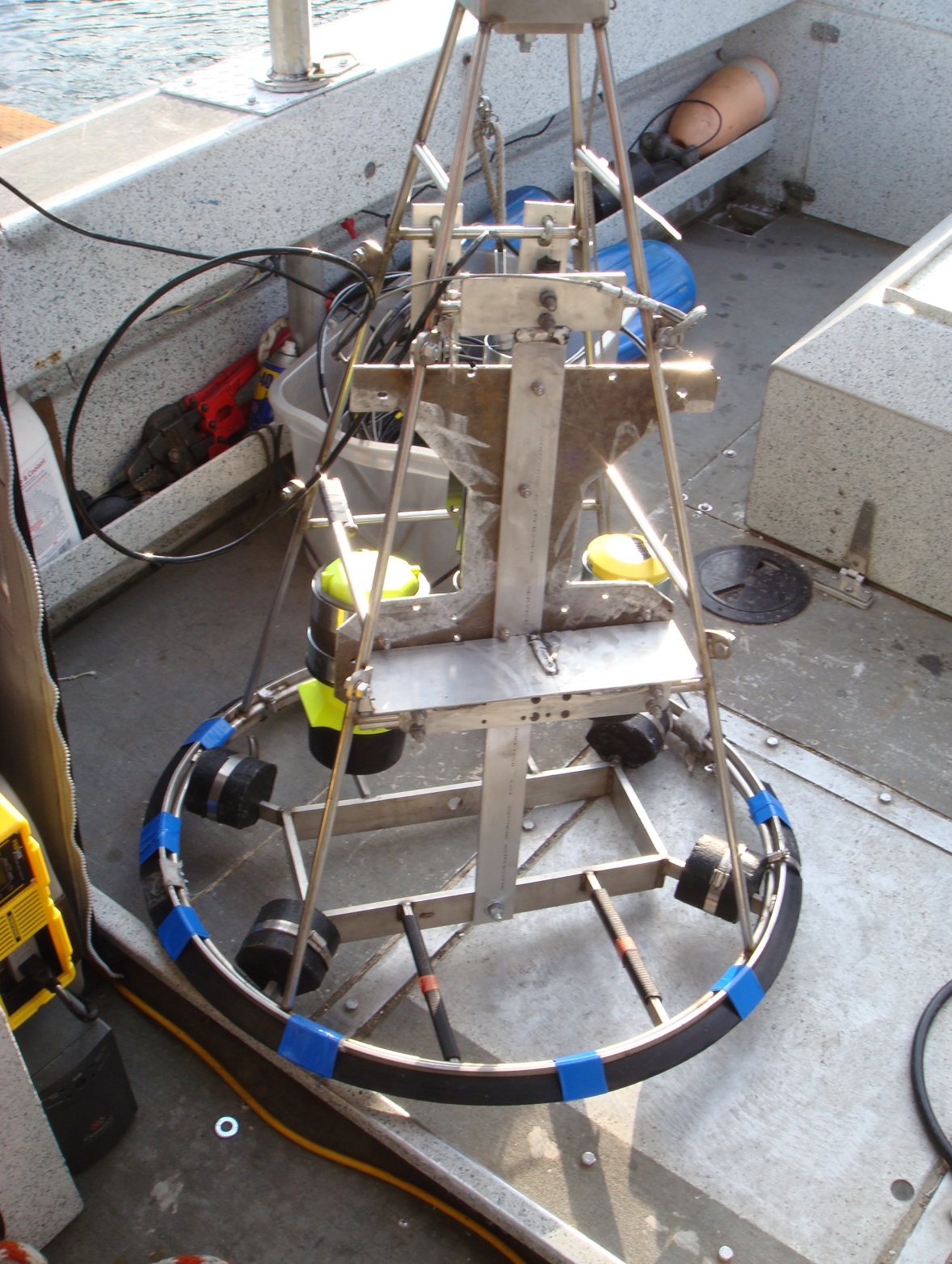


Figure A1.3. A modified grab-sampler equipped with a video camera to characterize substrate composition and embeddedness in three study reaches.

*A1.2.2. Substrate composition and embeddedness classification*

Using a video to JPEG convertor, we selected frames that had the clearest substrate image and imported them into image classification software (Media Cybernetics, 2001). Digitized classification techniques are less biased than Wolman pebble counts, provide more accurate particle sizes, and a larger quantity of measured particles (Whitman et al. 2003). With the reference view-frame captured on each image for scale (Fig. A1.4), we used the software to assist in the assignment of substrate particles to different size classes and to identify the dominant-subdominant particle classes (e.g., gravel/cobble, gravel/sand). This was accomplished by setting up a reference grid on the image of interest and using that calibrated grid to assign class sizes.

We used a modified Wentworth scale to place substrate particles into one of 10 size classes (Table A1.1.). During the substrate typing process, we used a relative embeddedness index to characterize the amount of embeddedness observed at each location (Whitman et al. 2003). Locations that had less than 5% embeddedness were assigned a rating of 0. Substrates that had no interstitial spaces, such as bedrock or cobble/gravels that were completely covered in sand, were assigned a rating of 5 (Table A1.2.). If the substrate was sand or fines without any other observable or sampled substrate underneath, then it was assigned an NA rating. In locations that we were unable to image the bottom due to extreme depths and current, we used our best professional judgment as to the substrate composition based upon the channel’s morphology,

Figure A1.4. ImagePro image of substrate in Skamania with a 2-cm grid overlaid. In this image, gravel and cobble are dominant and sub-dominant, respectively.


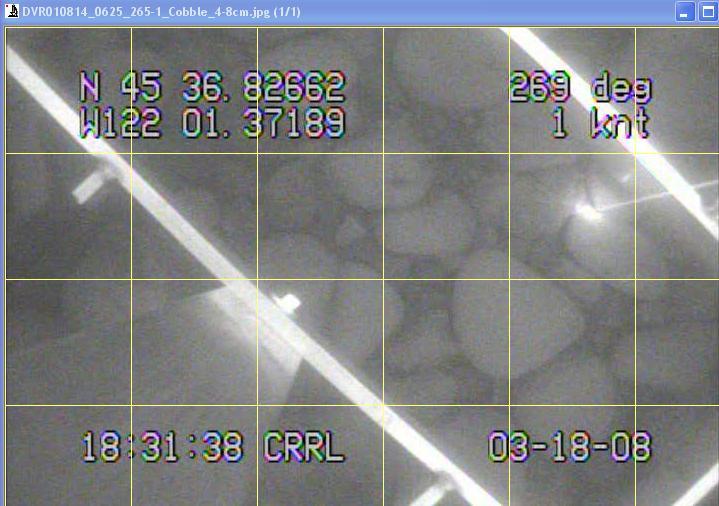


Table A1.1. Modified Wentworth substrate classification table.

| Substrate Classifications | Size |
| --- | --- |
| Impermeable ^a^ (bedrock, clay) |  |
| Boulder | >256 mm |
| Cobble | >64-256 mm |
| Coarse Gravel | >16-64 mm |
| Medium Gravel | >4-16 mm |
| Fine Gravel | 2-4 mm |
| Fines (sand/silt/mud) | < 2 mm |

Table A1.2. The relative composition of embeddedness found in six classes

^a^ not classified by particle size

| **Class** | **Embeddedness amounts** |
| --- | --- |
| 0 - Negligible | Coarse particles have <5% of surface covered by fines |
| 1 - Low | Coarse particles have 6-25% of surface covered by fines |
| 2 - Moderate | Coarse particles have 26-50% of surface covered by fines |
| 3 - High | Coarse particles have 51-75% of surface covered by fines |
| 4 - Very High | Coarse particles have >75% of surface covered by fines |
| 5 - N/A | Fine grained sediments w/o gravel or cobble |

surrounding substrate, and velocities. For instance, we rationalized that areas of high velocities (>2 m/sec) would be comprised of large boulders or bedrock based upon stream power and sediment ratios.

In addition to the still-frame video imaging technique to determine embeddedness, we viewed live video feed of the sediment plume created when the grab-sampler contacted the bottom. If a sediment plume produced little to no plume, embeddedness was usually very small. Plumes that took ~5 – 10 seconds to disperse usually had a moderate amount of embeddedness. Large sediment plumes that lingered for greater than 10 seconds usually indicated heavy embeddedness. We created GIS sediment and embeddedness maps by digitizing sampling locations of like type (e.g., low embeddedness, cobble substrate). We smoothed the boundaries so they had a more natural shape (e.g., parallel to flow), versus boundaries with sharp or acute angles.

*A1.2.3. Accuracy assessment*

We randomly selected 100 points from the Skamania and John Day reaches and collected new video samples for verification purposes, processing video samples using the same techniques as before. We overlaid the verification samples on the substrate composition and embeddedness maps (i.e., grids) with GIS and calculated the agreement between the two products. For example, if 7 out of 10 map cells were classified similarly to the reference locations, the accuracy would be 70%. In the Kootenai reach, numerous field projects were conducted prior to, simultaneously, or immediately after our efforts, which contributed extensive underwater videography, sediment cores, and multibeam sonar data (Barton et al. 2005; McDonald et al. 2010). The quantity and quality of data supplemented our mapping efforts and produced the highest-resolution substrate maps of the three reaches. Given the extensive editing and refinement of the Kootenai substrate map, for all practical purposes it is considered to be essentially 100% accurate.

**A1.3. Results**

*A1.3.1. Substrate Composition*

The substrates of the three reaches varied substantially. The Skamania reach was comprised predominantly of gravel (53%) and fines (28%), with lesser amounts of boulder and bedrock (Fig. A1.5A, B). In addition, approximately 4% of the substrate was comprised of shell hash resulting from large quantities of freshwater clams, with the remaining area (~3%) comprised of island. In the John Day reach, substrate was comprised predominantly of gravel (62%) and cobble (22%) (Fig. A1.6A, B) with lesser amounts of fines, boulder/bedrock, clay, and shell hash. In both the Skamania and John Day reaches, coarser-size substrates were more often found in the upstream end where velocities were greatest, with smaller cobble and gravels located in the middle and lower portions of the reach. Fines were largely found along the lateral margins where velocities and stream power were reduced. In the Kootenai reach, fines comprised 81%, gravel 11%, and clay 6.5% (Fig. A1.7). Thus, the primary difference between the reaches was the fraction of fines versus gravel (Fig.A1.8); the Skamania and John Day reaches were comprised largely of gravels and cobbles, while Kootenai reach was largely sands and fines.

*A1.3.2. Embeddedness*

The embeddedness of each reach varied considerably. In the Skamania reach (Fig. A1.9A, B), 58% of the substrate had low embeddedness (<25% fines), 33% was functionally classified as completely embedded (i.e., fines or bedrock), and the remaining 9% of the reach

had moderate (6.6%) to high embeddedness (2.4%). In the John Day reach (Fig. A1.10A, B), 83% of the substrate had low embeddedness, 8.6% was functionally completely embedded, and the remaining 8.4% moderately embedded. In the Kootenai reach (Fig. A1.11A, B), approximately 5% had low embeddedness, 82% was functionally completely embedded (fines), and the remaining 13% a mixture of moderate to high embeddedness. Collectively, the Skamania and Upper John Day reaches are the mirror image of the Kootenai reach (Fig. A1.12), with their largest embeddedness class on the lowest end of the spectrum (< 5%) and Kootenai at the highest end (completely embedded).


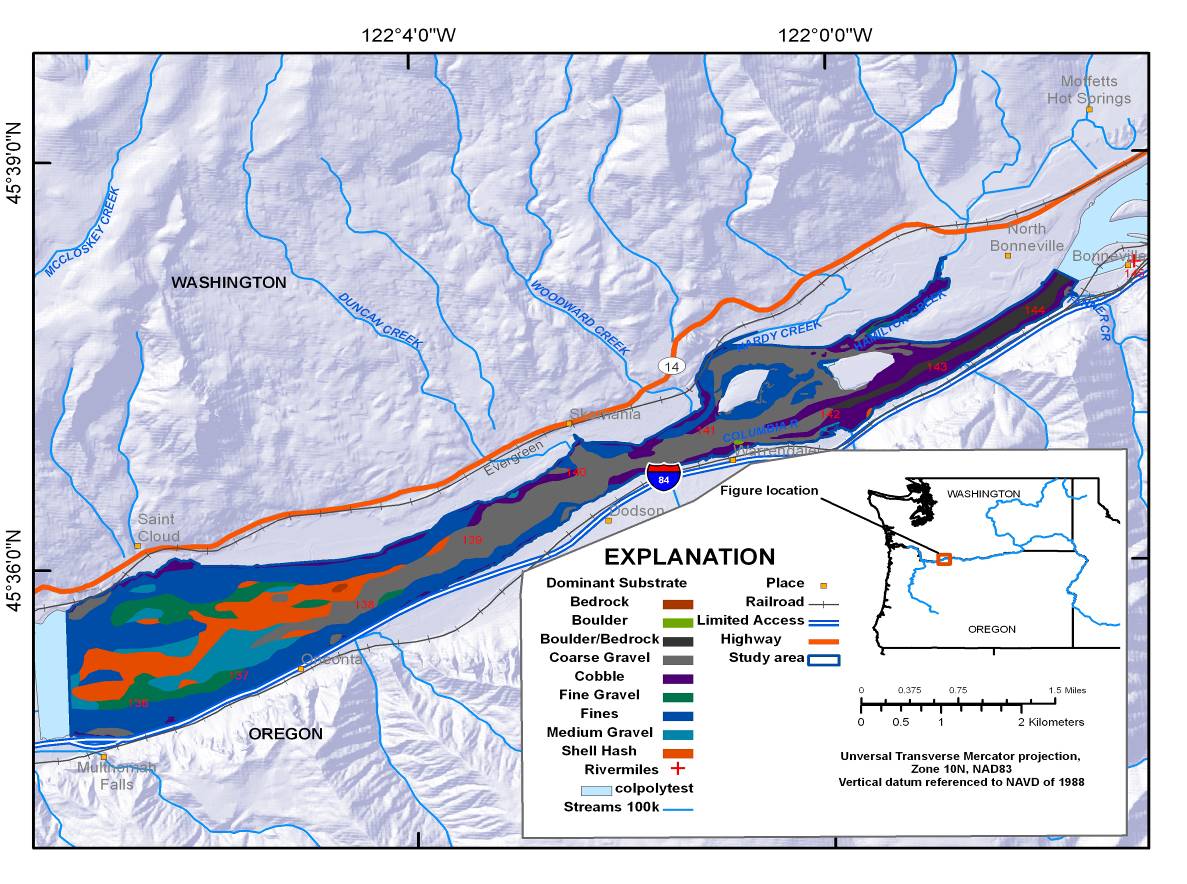


Figure A1.5. A substrate map of the Skamania Reach (top panel) and a bar graph depicting the amount of area (%) that each substrate class comprised in Skamania reach (bottom panel).


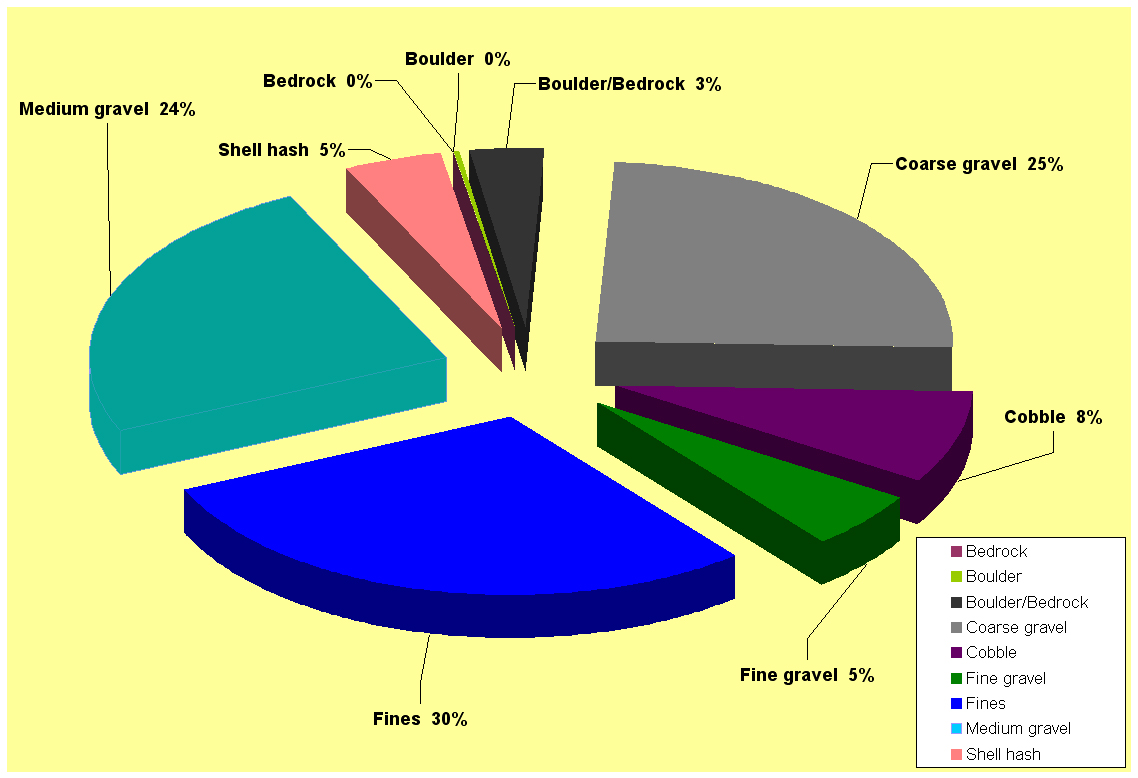

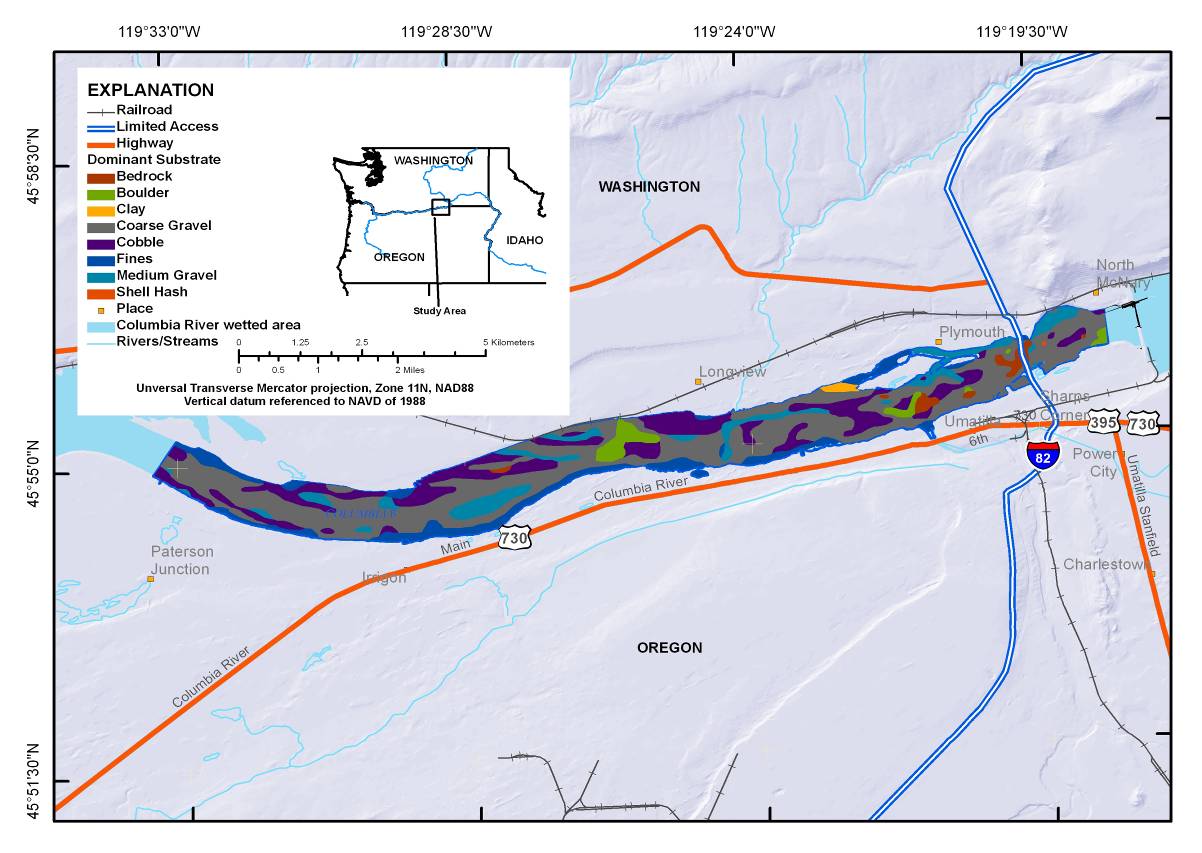


Figure A1.6. A substrate map of the Upper John Day Reach (top panel) and a pie chart (bottom panel) displaying the area (%) that each substrate class comprised in the Upper John Day reach.


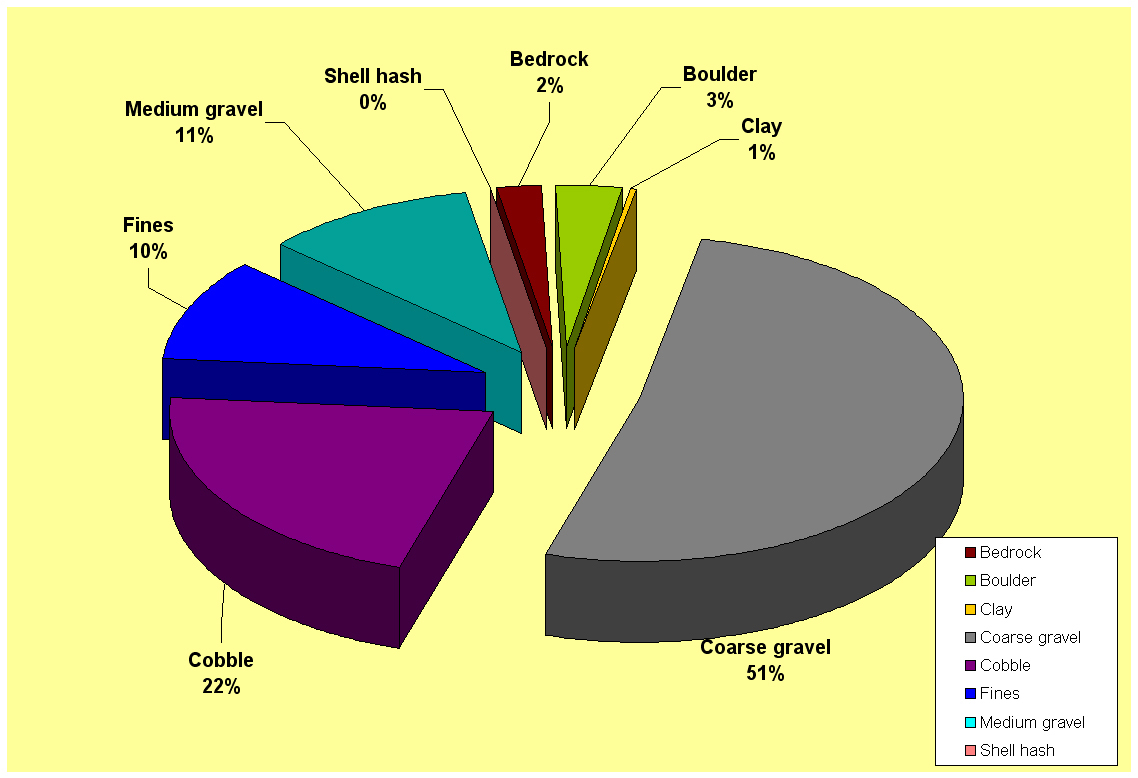

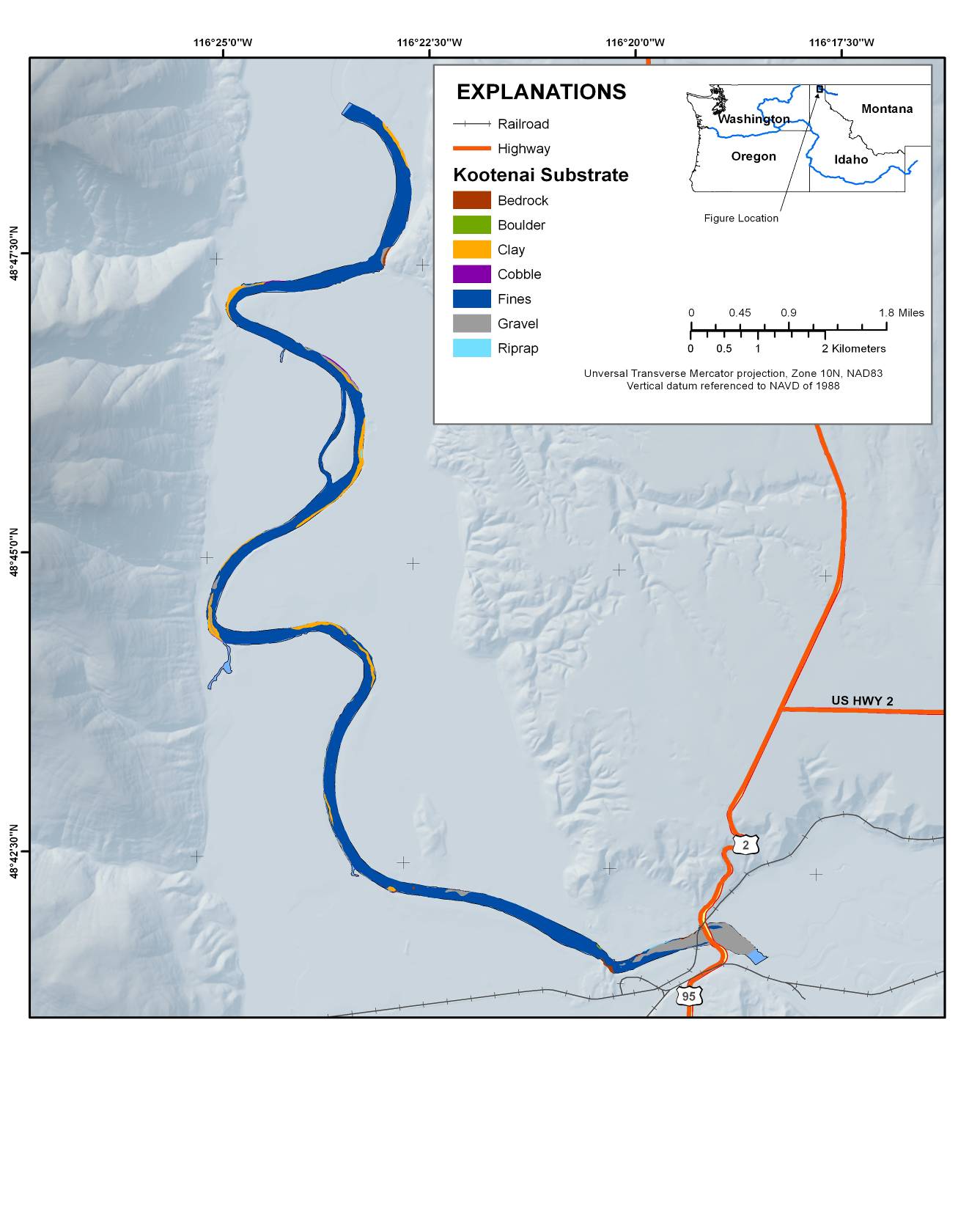


Figure A1.7. A substrate map of the Kootenai (Meander) reach and a pie chart (map inset) that depicts the area (%) that each substrate class comprised in the Kootenai reach.


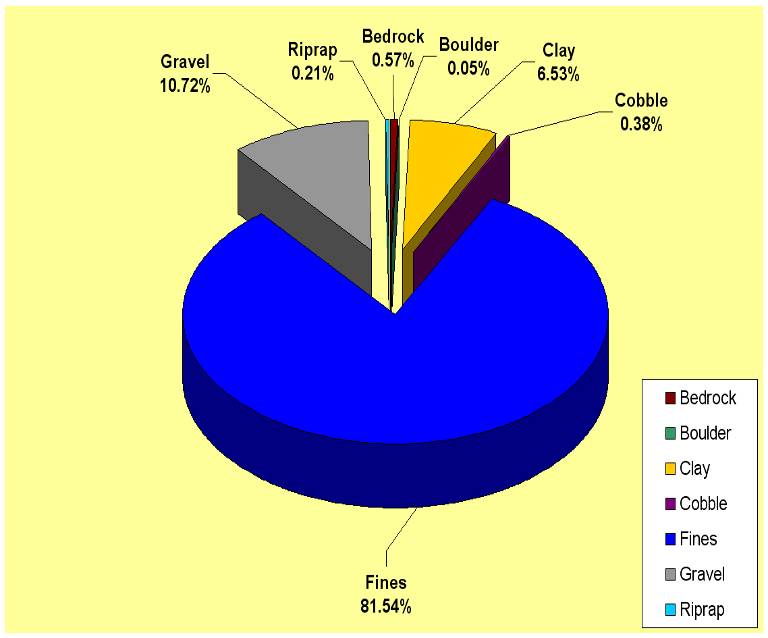


Figure A1.8. The relative amounts (percent) that each substrate class comprised in the study reaches.


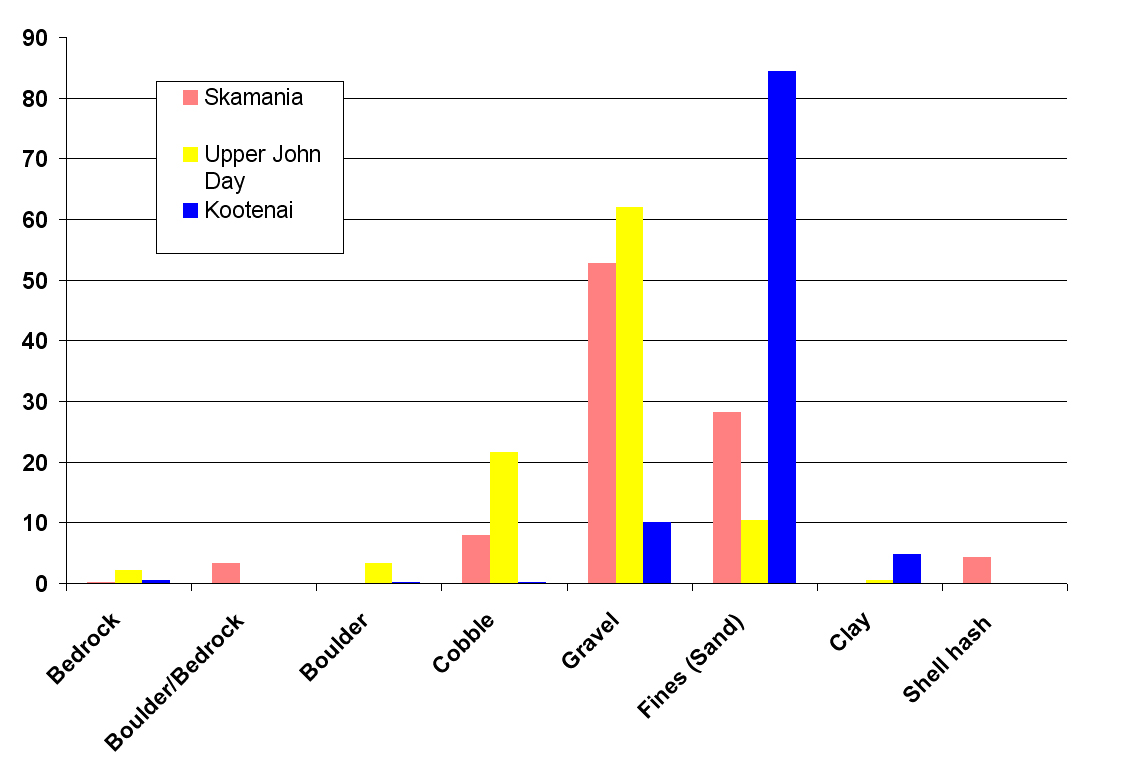

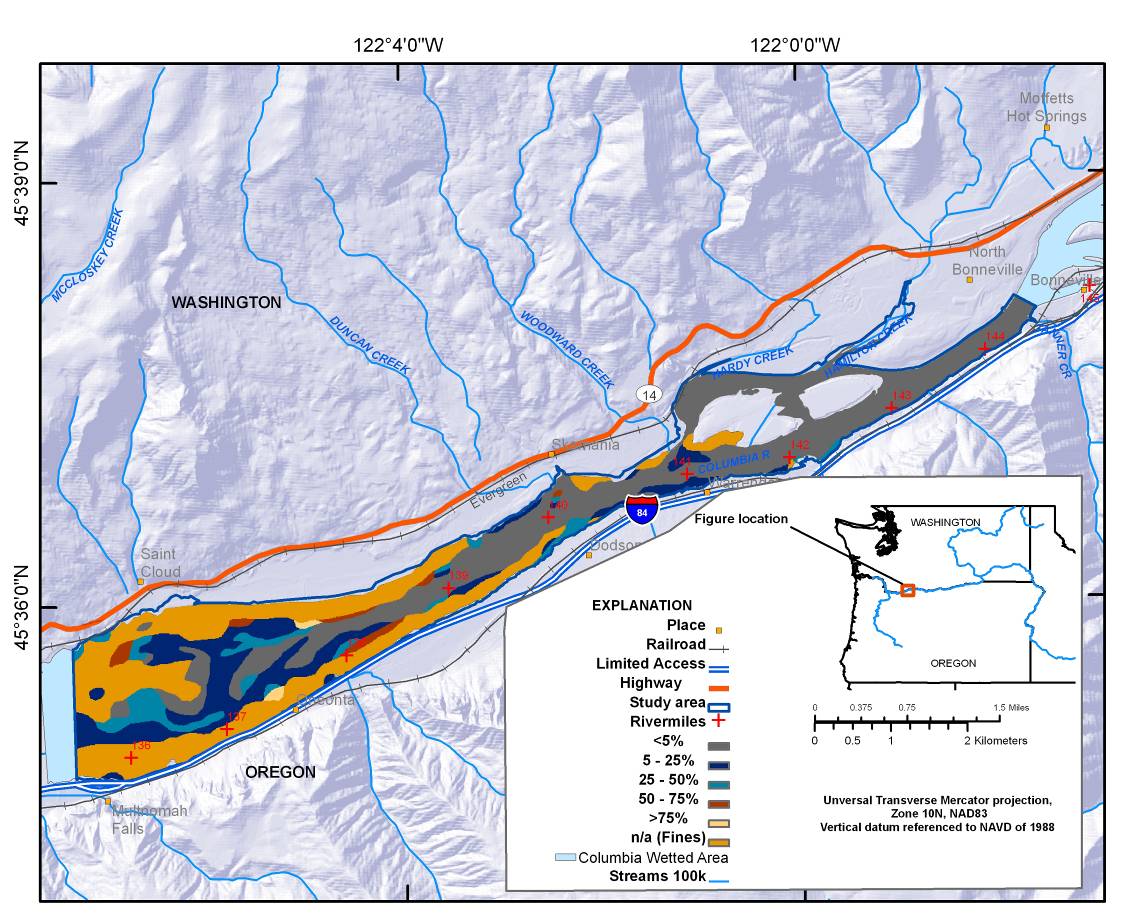


**Figure A1.9. An embeddedness map of the Skamania reach (top panel), and a pie graph depicting the amount (ha) and area (%) that each embeddedness class comprised in Skamania reach (bottom panel).**


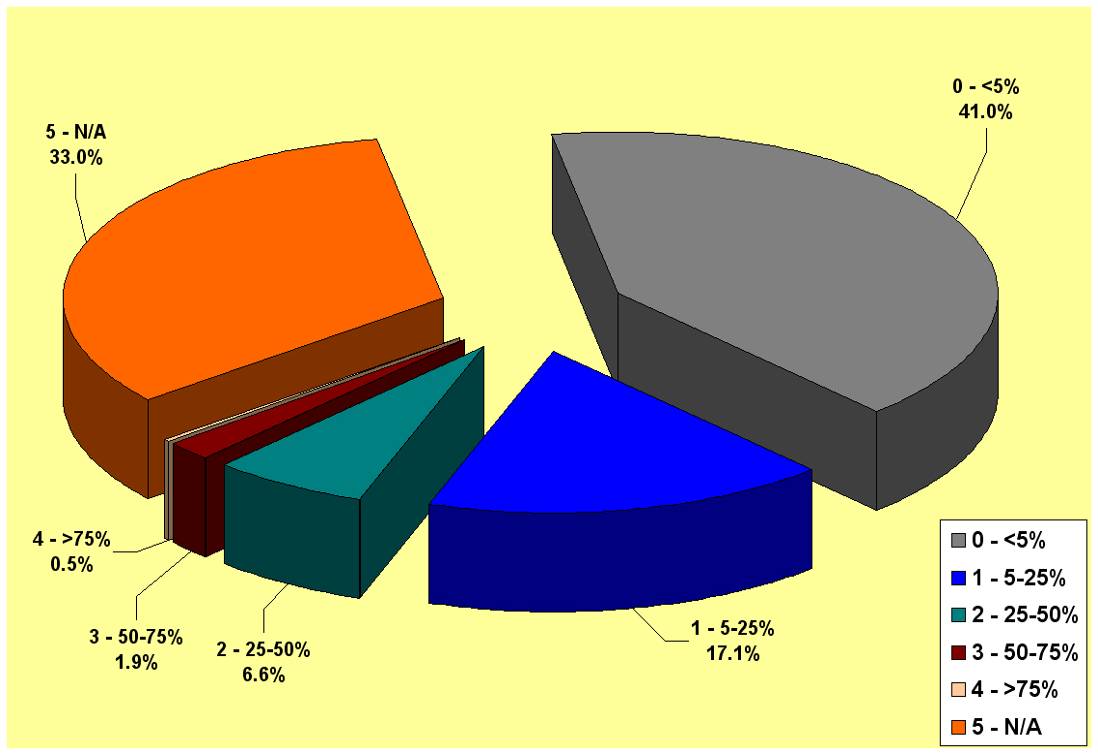


*A1.3.3. Classification Accuracy*

In the Skamania reach, there is a large section of the thalweg between river kilometers 229 and 232 where the currents are greater than 2 m/sec, and depths greater than 26 meters, which prevented us from safely capturing video footage of the river bottom. Given the extreme velocities (3 - 3.5 m/sec), channel morphology and surrounding substrate composition, we classified this reach as bedrock, with an effective embeddedness rating of five (i.e., no interstitial spaces). In the John Day reach, between river kilometers 456 and 459, there is a large gravel bar on the southern half of the river that we were unable to safely navigate in a boat due to the shallow water. In that section, we waded and visually estimated the substrate composition and embeddedness. Most of the bar we classified as medium gravel with a moderate embeddedness rating. Due to high currents and difficult lighting conditions, only 77 samples were used in accuracy assessment of Skamania reach, and 81 samples in the John Day reach. When compared to validation data, we achieved 83% accuracy in substrate mapping in Skamania reach and 80% in John Day. Related to embeddedness, we achieved 70% accuracy in the Skamania reach and 80% in the Upper John Day.

**A1.4. Discussion**

The Kootenai reach is very different from the other two study reaches in several ways.


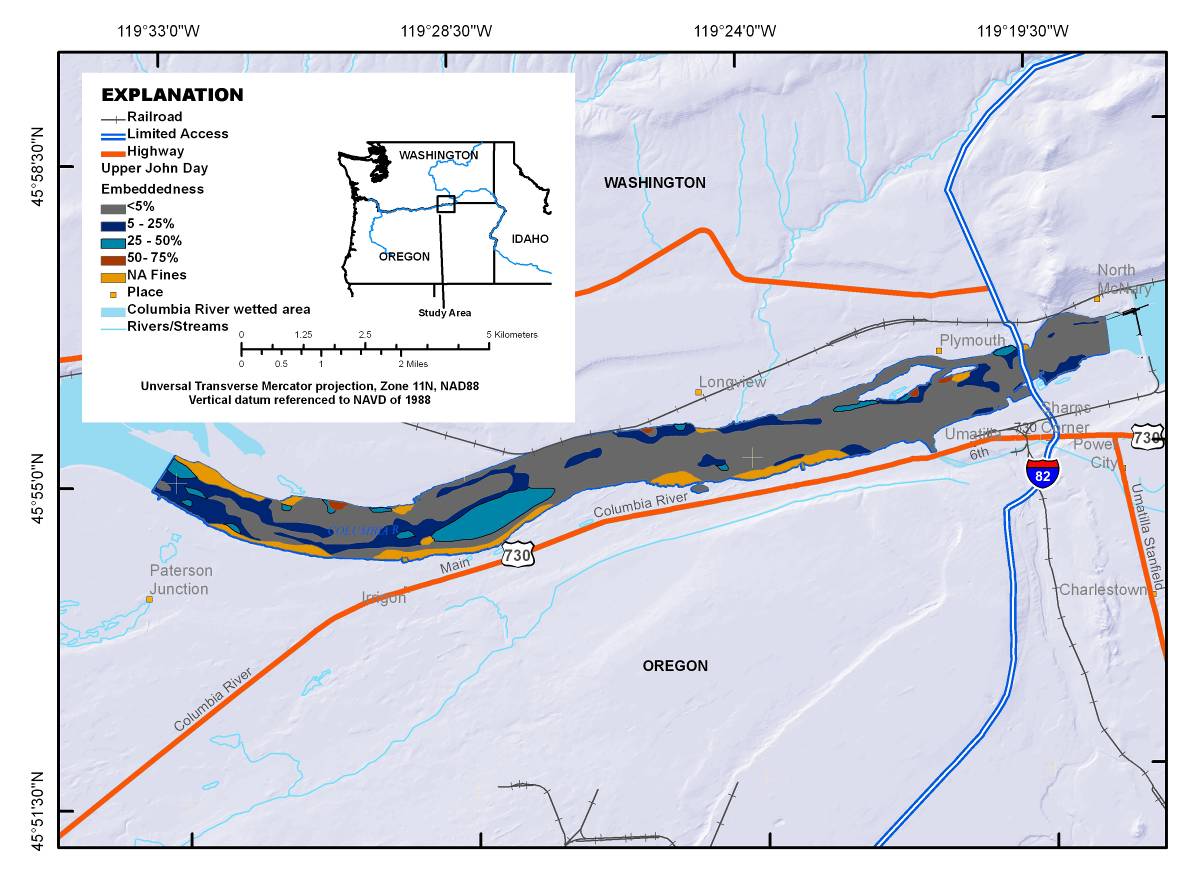


**Figure A1.10. An embeddedness map of the John Day reach (top panel), and a pie graph depicting the amount of area (%) that each embeddedness class comprised in the John Day reach (bottom panel).**


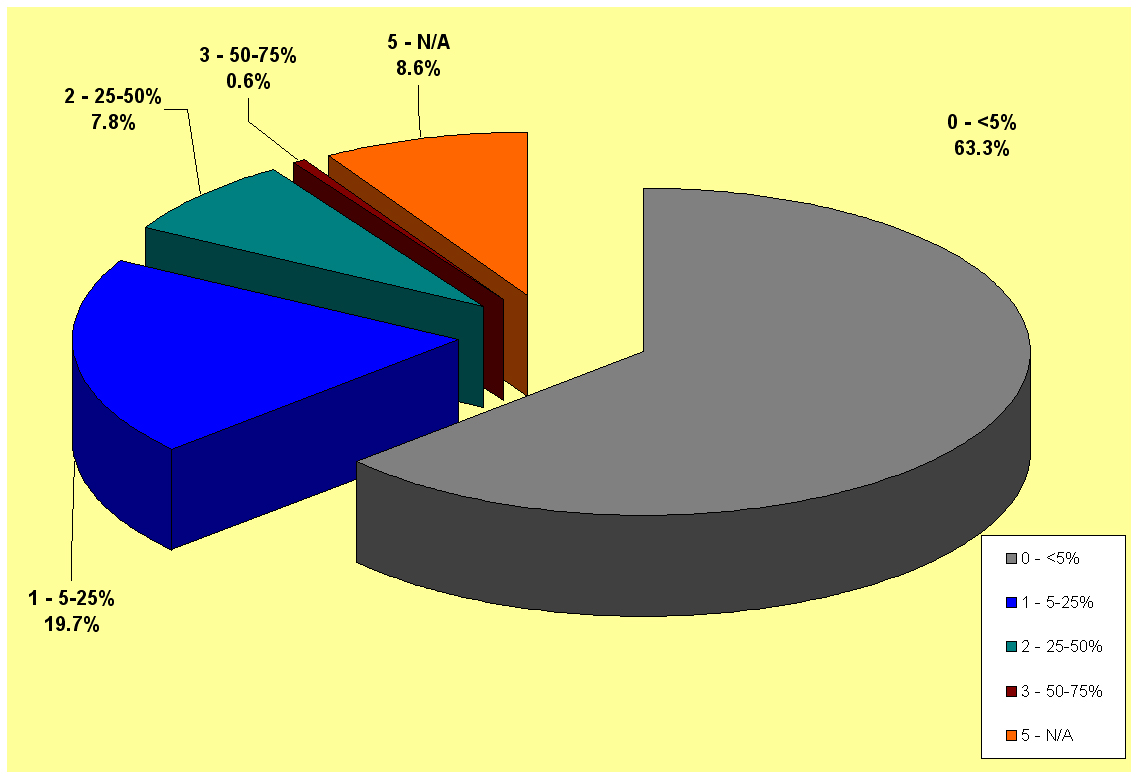


**Figure A1.11. An embeddedness map of the Kootenai (Meander) reach, and a pie chart (inset) that depicts the amount of area (%) that each embeddedness class comprised.**


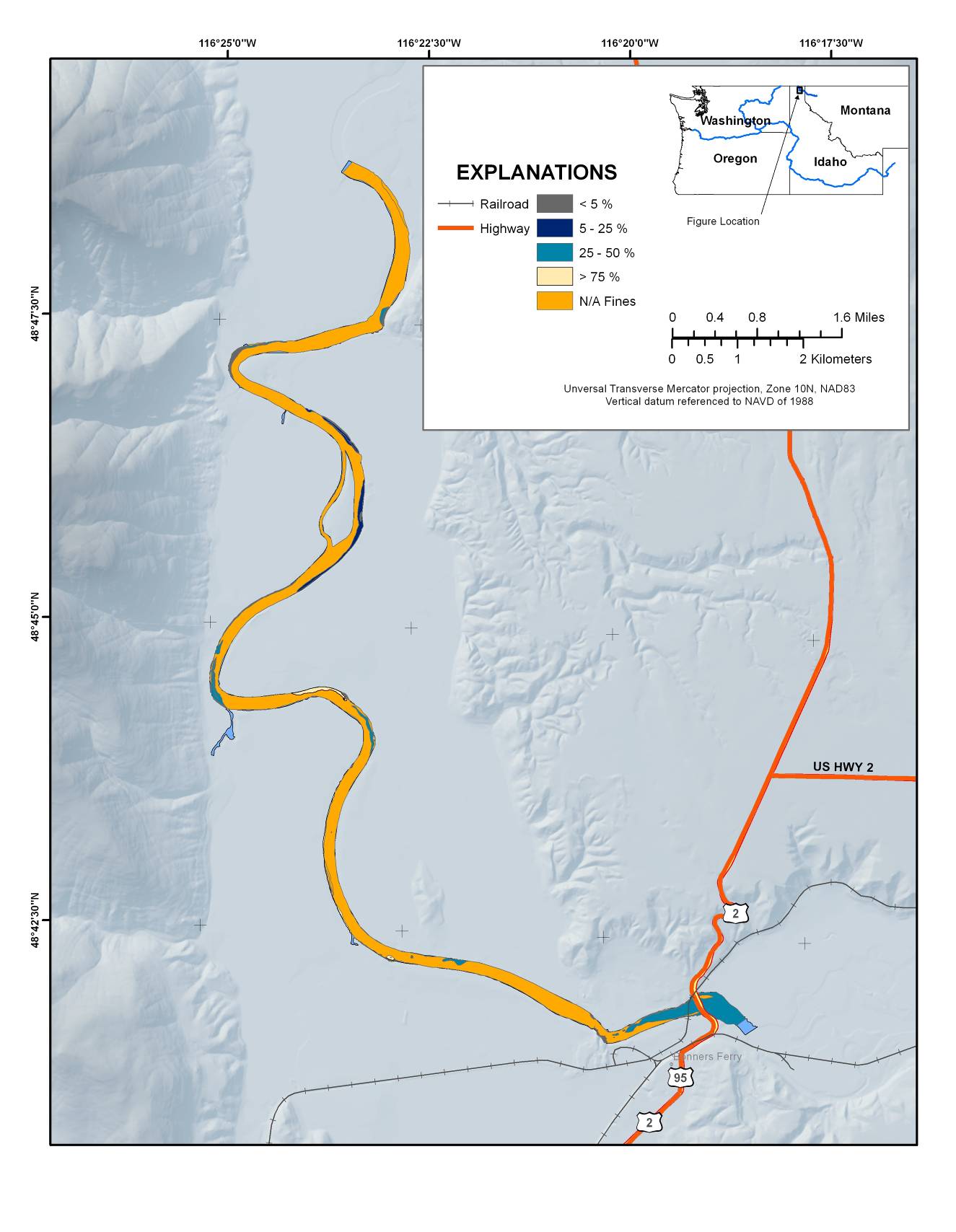

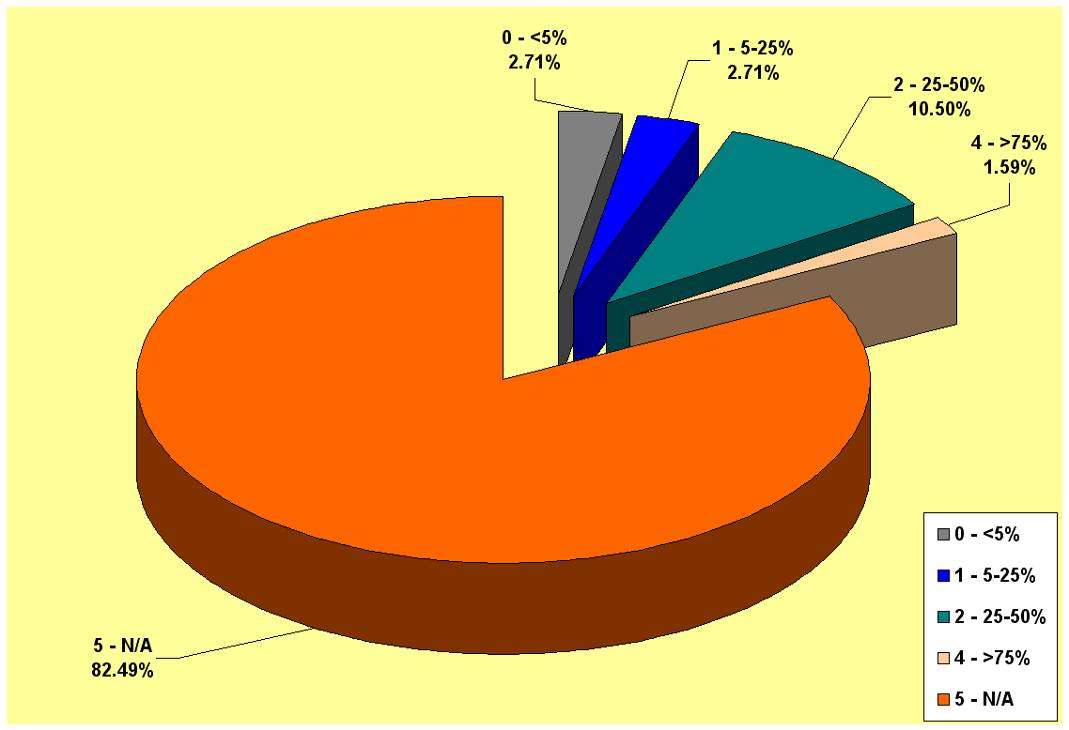


**Figure A1.12. The proportion of area that each embeddedness class (1 – 6) comprised in the three study reaches**.


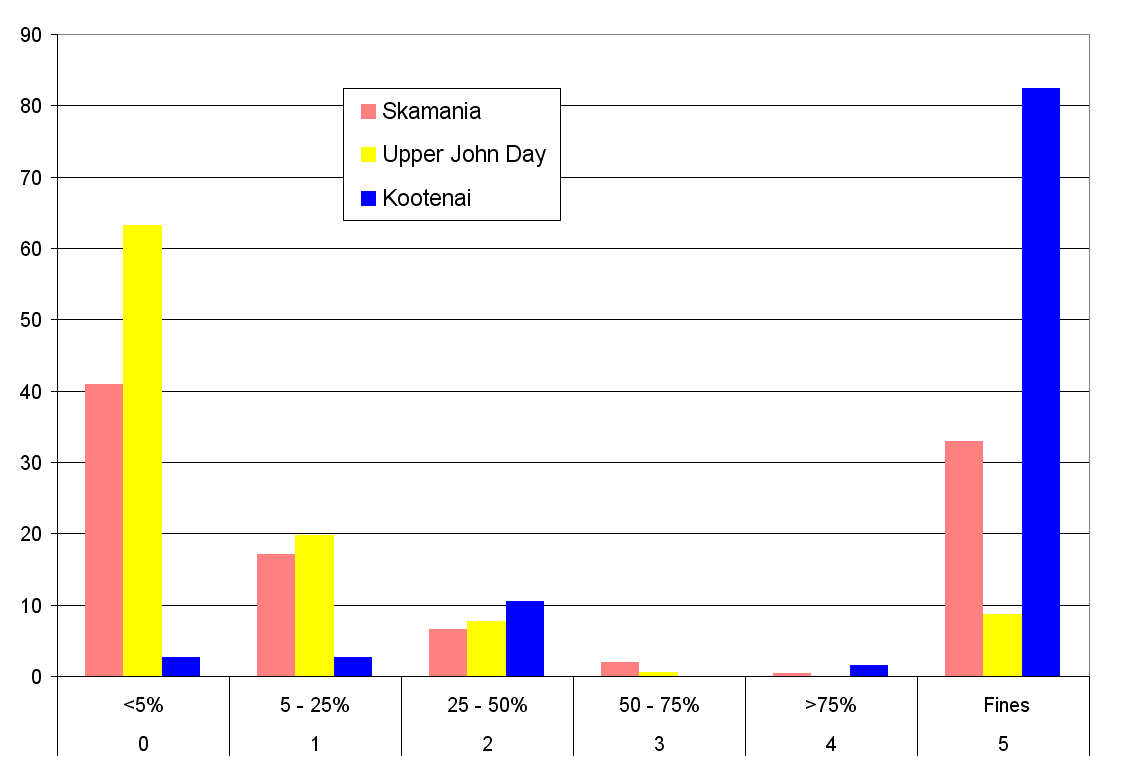


First, its shape is long, sinuous, and narrow. Second, its bottom is comprised largely of fine

sands. Third, it is functionally embedded since sand covers most of the river’s bottom. In contrast, the Skamania reach is very diverse in terms of its hydraulics, substrate composition, and embeddedness patterns. With its deep, swift chute that emanates from the Bonneville Dam tailrace, its islands and side-channels, it is the most diverse of the three study reaches. In contrast, the John Day reach, which is located in the middle of the three reaches, has some unique physical qualities. First, it has the largest concentration of gravels and cobbles and the lowest embeddedness levels. Second, it lacks the deep chute that Skamania reach has, or the long, sinuous pattern of the Kootenai reach. Third, it is backwatered from the John Day Dam, giving it a fatter shape than the other two reaches.

Most of the classification errors associated with our substrate and embeddedness maps are attributable to variable lighting conditions resulting from deep, swift, turbid waters. It is likely that higher-resolution video cameras and improved lighting would improve our classification (Rooper, 2008). High-resolution still images would allow us to train the video/image processing software to automate substrate mapping. Furthermore, the use of a multibeam sonar system would allow us to more accurately classify larger areas of the river’s bottom, rather than the interpolation techniques that we used from spot samples. Nonetheless, our substrate composition and embeddedness maps are the most complete to date for the three study reaches and provide significant information.

**References not listed in main document**

Garland, R.D, Rondorf, D.W., and Tiffan, K.F. 2003. Assessment of Chum and Fall Chinook Salmon Spawning Habitat Near Ives and Pierce Island In The Columbia River. Annual Report 1999-2001.

Media Cybernetics, Inc. Image-Pro Plus Version 4.5 for Windows Auto-Pro Reference.

Software Manual 2001.

Rooper, C.N. 2008. Underwater Video Sleds: Versatile and cost Effective Tool for Habitat Mapping. Marine Habitat Mapping Technology for Alaska, Alaska Sea Grant College Program, University of Alaska Fairbanks. CD-ROM doi:10-4027/mhmta 2008.07.

Whitman. M.S., Moran, E.H.,and R. T. Ourso. 2003. Photographic Techniques for Characterizing Streambed Particle Sizes. Transactions of the American Fisheries Society 132:605–610.
