# Supplementary material for: Substrate and flow characteristics associated with White Sturgeon recruitment in the Columbia River Basin
Source: Heliyon. 2018 May 21;4(5):e00629. doi: 10.1016/j.heliyon.2018.e00629 (PMC5986543; doi:10.1016/j.heliyon.2018.e00629)
Supplement: Appendix 2. Substrate Froude and embeddedness maps [file mmc2.docx]

**Appendix 2. Substrate, Froude, and embeddedness maps**


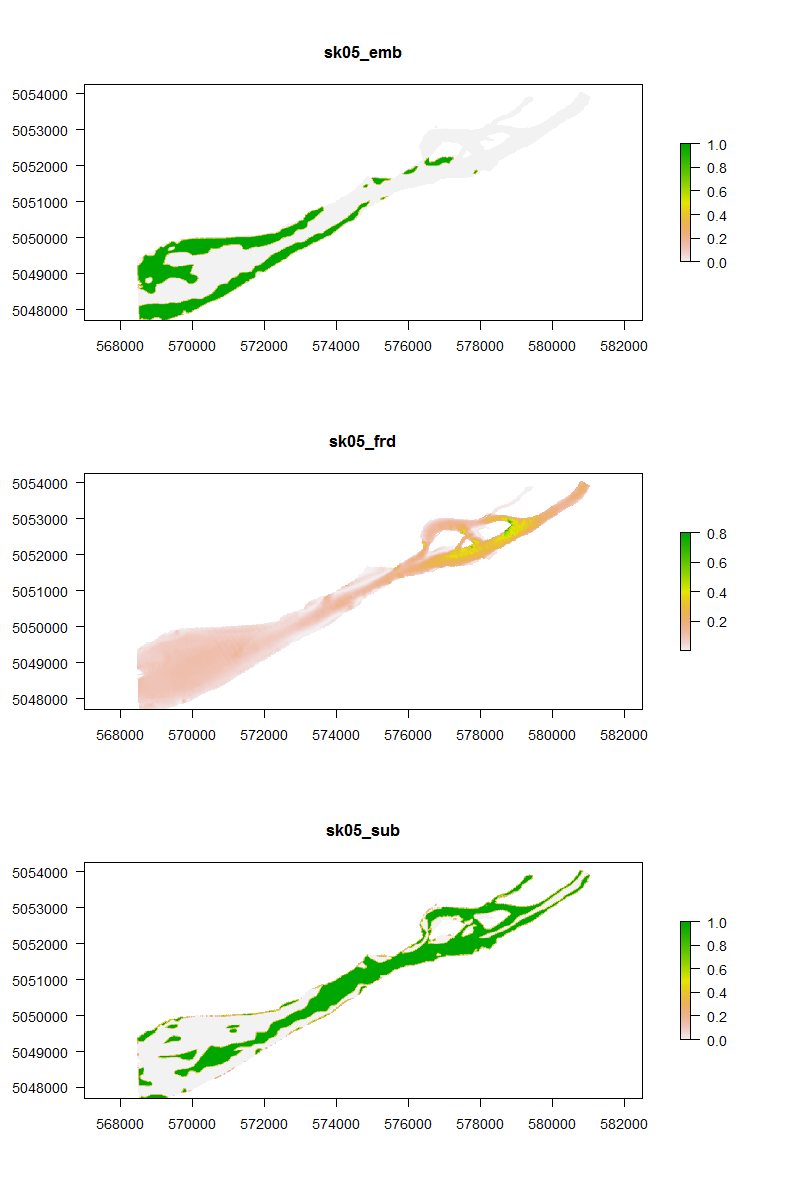


Fig. A2.1. Maps of embeddedness levels (sk05_emb), Froude numbers (sk05_frd), and gravel/cobble compositions (sk05_sub) inside Skamania reach at a 5% exceedance flow. Coordinates are UTM, Zone 10, NAD 83.


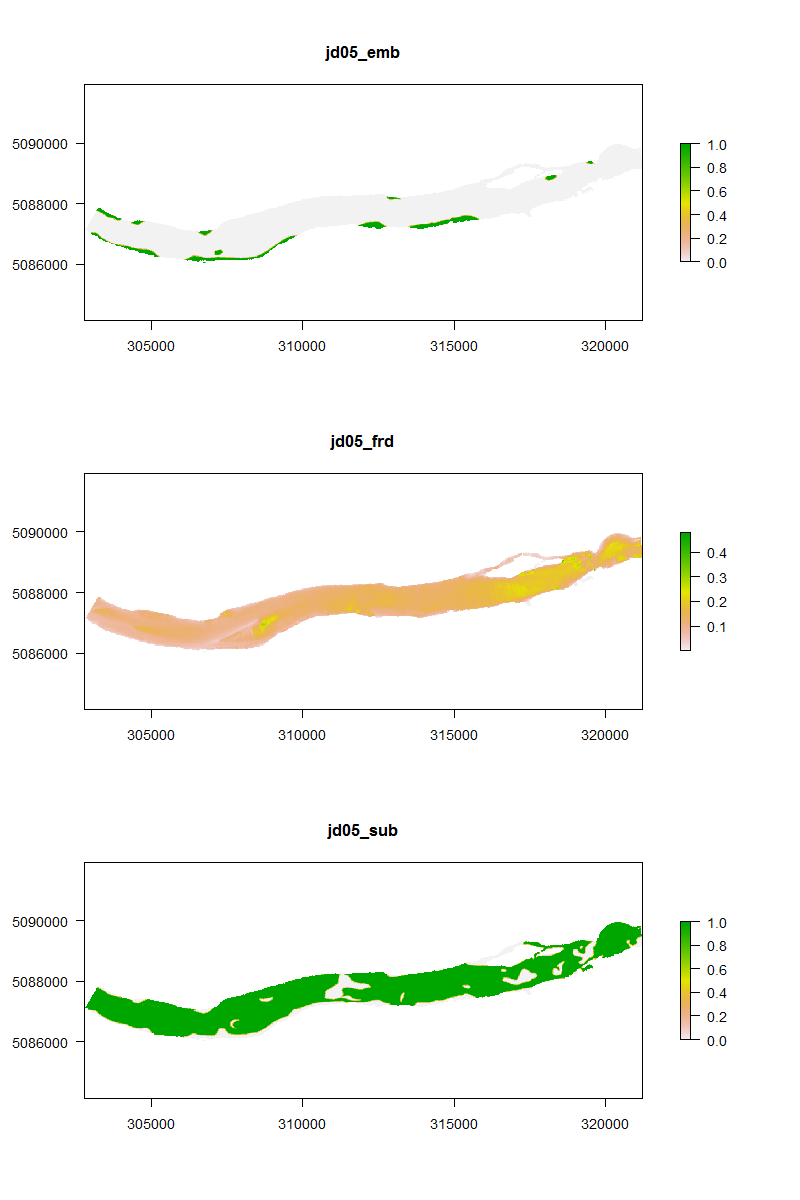


Fig. A2.2. Maps of embeddedness levels (jd05_emb), Froude numbers (jd05_frd), and gravel/cobble compositions (jd05_sub) inside John Day reach at a 5% exceedance flow. Coordinates are UTM, Zone 11, NAD 83.


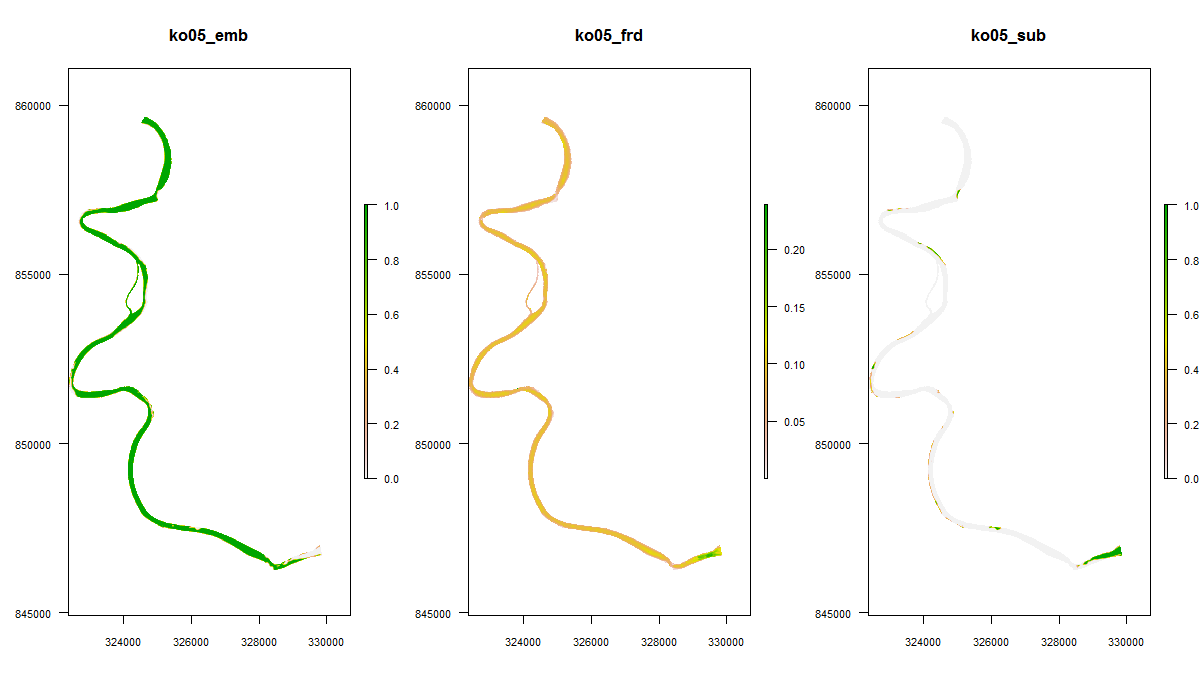


Fig. A2.3. Maps of embeddedness levels (ko05_emb), Froude numbers (ko05_frd), and gravel/cobble compositions (ko05_sub) inside Kootenai reach at a 5% exceedance flow. Coordinates are Transverse, NAD 83.
