# Supplementary material for: Substrate and flow characteristics associated with White Sturgeon recruitment in the Columbia River Basin
Source: Heliyon. 2018 May 21;4(5):e00629. doi: 10.1016/j.heliyon.2018.e00629 (PMC5986543; doi:10.1016/j.heliyon.2018.e00629)
Supplement: Appendix 3. Model outputs [file mmc3.docx]

**Appendix 3. Model outputs**


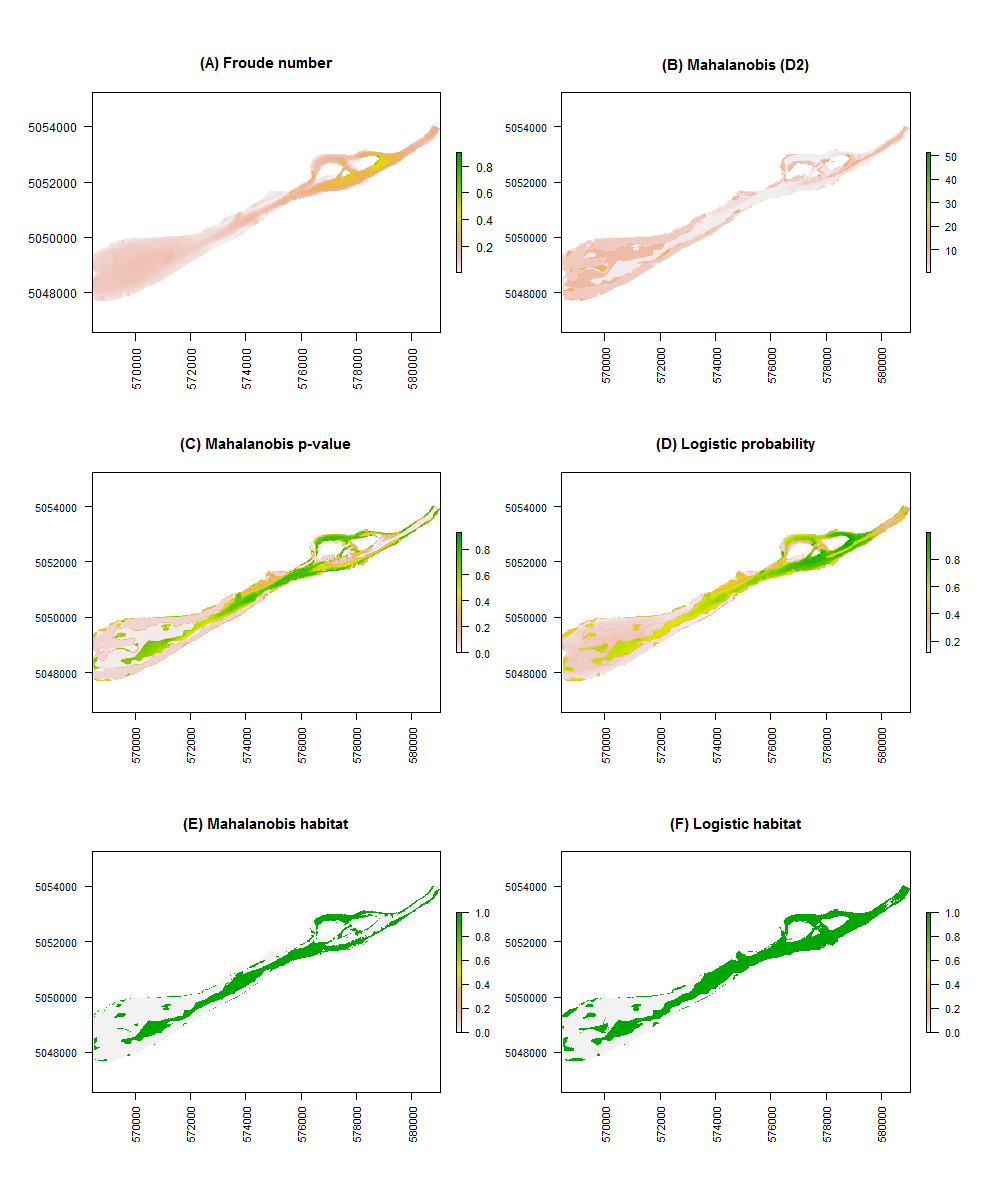


Fig. A3.1. Maps of Froude number (A), Mahalanobis distance (B), Mahalanobis Chi-square p-value (C), logistic regression probability (D), Mahalanobis habitat suitability map (E), and logistic habitat suitability map (F) inside Skamania reach at a 5% exceedance flow. Coordinates are UTM, Zone 10, NAD 83.


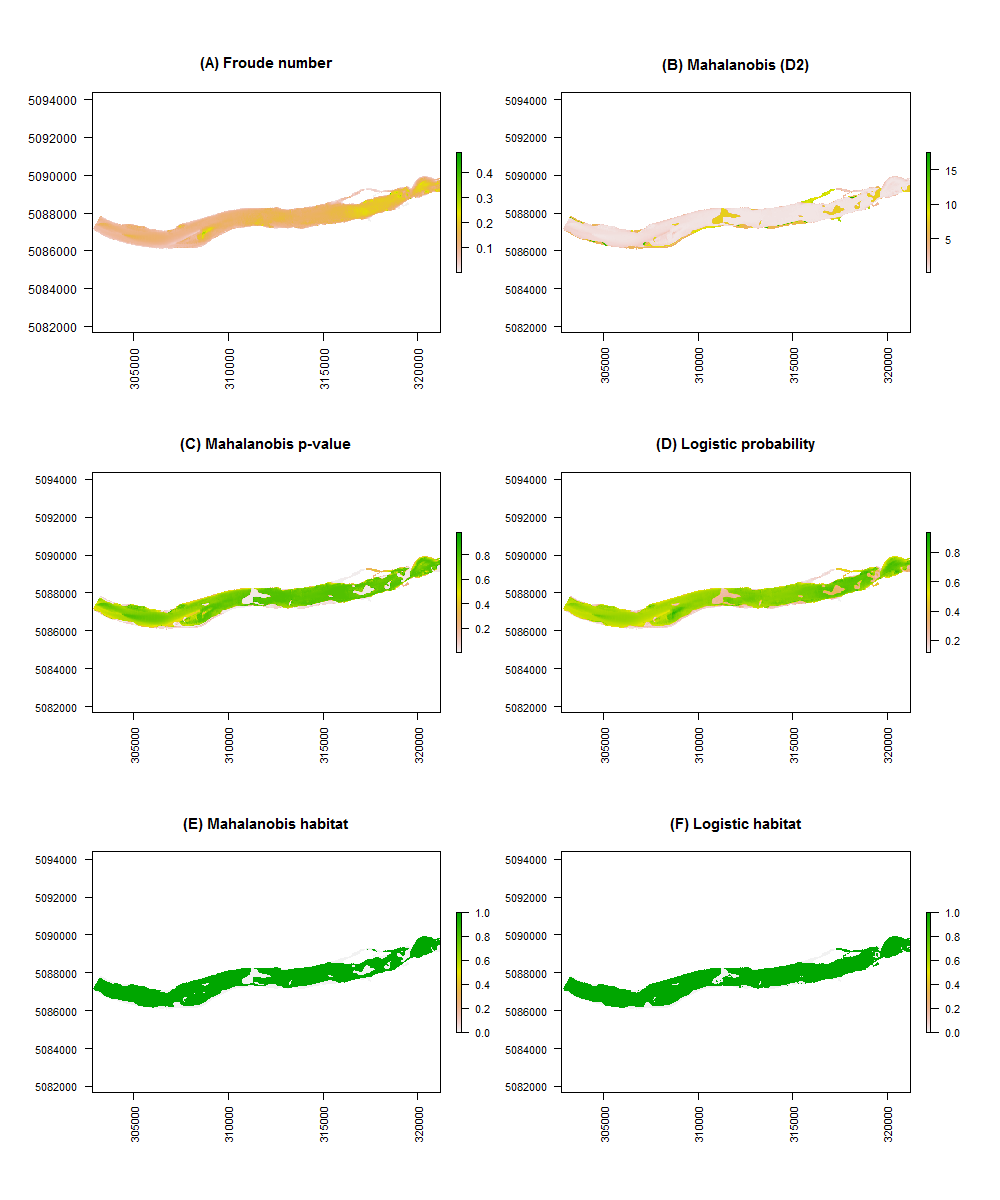


Fig. A3.2. Maps of Froude number (A), Mahalanobis distance (B), Mahalanobis Chi-square p-value (C), logistic regression probability (D), Mahalanobis habitat suitability map (E), and logistic habitat suitability map (F) inside John Day reach at a 5% exceedance flow. Coordinates are UTM, Zone 11, NAD 83.


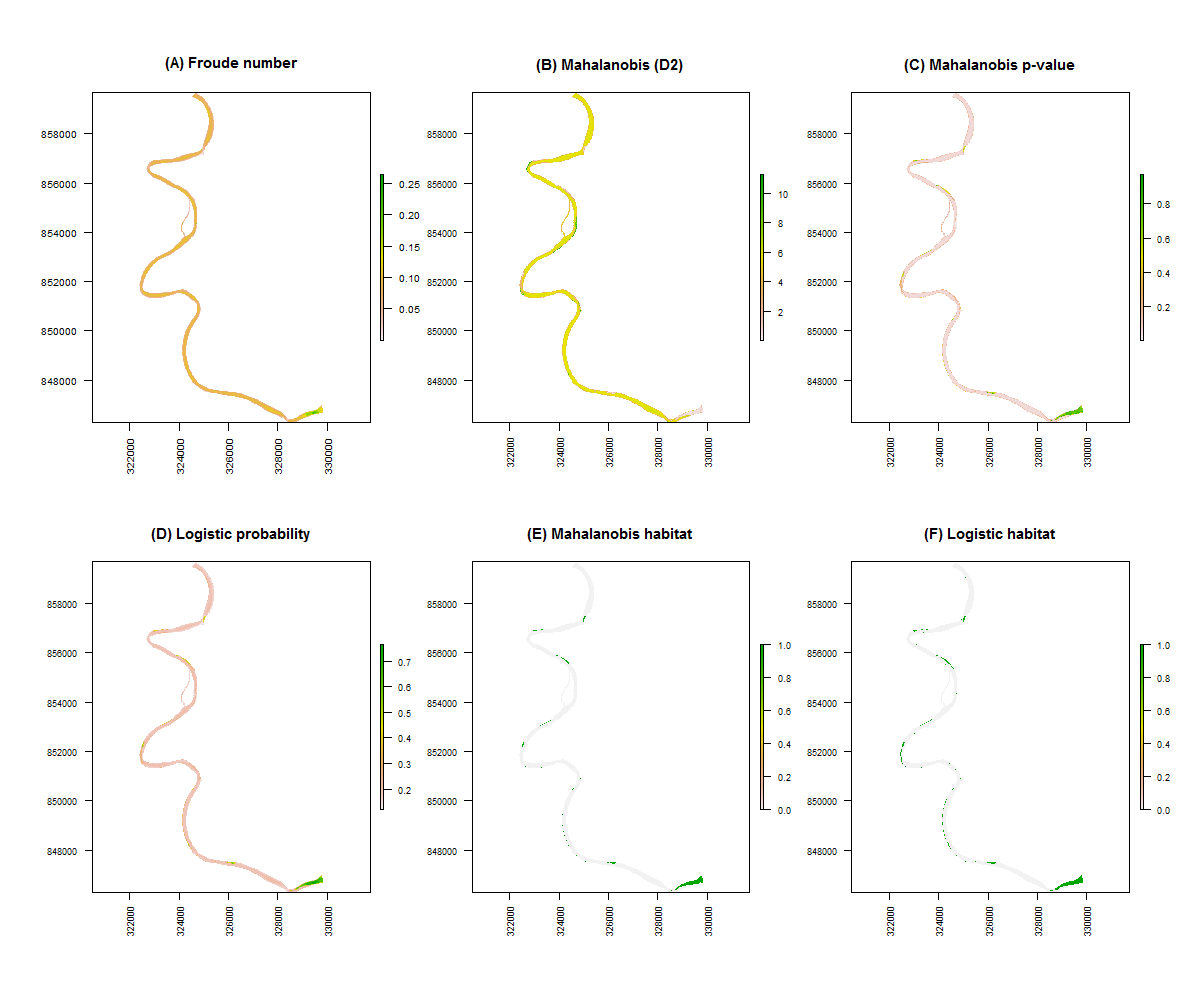


Fig. A3.3. Maps of Froude number (A), Mahalanobis distance (B), Mahalanobis Chi-square p-value (C), logistic regression probability (D), Mahalanobis habitat suitability map (E), and logistic habitat suitability map (F) inside Kootenai reach at a 5% exceedance flow. Coordinates are Transverse, NAD 83.
